# Functional assessment of CELSR1 variants associated with human developmental defects

**DOI:** 10.3389/fcell.2026.1818587

**Published:** 2026-10-08

**Authors:** Jazmin M. Stenson, William Giang, Sarah G. Latario, Courtney L. Williams, Valerie Trujillo Adorno, Congzhou M. Sha, Sara N. Stahley

**Affiliations:** 1 Department of Dermatology Penn State College of Medicine, Hershey, PA, United States; 2 Biomedical Sciences Graduate Program Penn State College of Medicine, Hershey, PA, United States; 3 Medical Scientist Training Program, Penn State College of Medicine, Hershey, PA, United States; 4 Department of Cell and Biological Systems Penn State College of Medicine, Hershey, PA, United States

**Keywords:** cadherin, cell adhesion, CELSR1, congenital heart defect, neural tube defect, PCP, planar cell polarity, variant pathogenicity

## Abstract

During development, collective and oriented cell behaviors that drive organ formation and tissue patterning require coordination among neighboring cells governed by the highly conserved planar cell polarity (PCP) pathway. Deficiencies in PCP gene function result in severe developmental defects in model systems from flies to mice, while PCP gene mutations are associated with human birth defects impacting the neural tube and heart. Atypical cadherin Celsr1 engages in cell-cell adhesion to coordinate the asymmetric localization of Fz6 and Vangl2 to opposing sides of the PCP junction between neighboring cells. However, whether *CELSR1* genetic variations disrupt function or contribute to disease pathogenesis remains poorly understood. Using cell-based assays for Celsr1 function, along with *in silico* modeling, we test a cohort of 17 Celsr1 mutations previously identified in patients with neural tube and congenital heart defects. In junctional enrichment assays, we find 13 variants displayed altered enrichment at cell borders relative to WT, including both loss- and gain-of-function behaviors. Further analysis of select Celsr1 mutations revealed defects in membrane mobility and perturbed co-recruitment of PCP protein Fz6. Our findings link Celsr1 mutations associated with human birth defects to functional defects in adhesion and PCP. In addition, our approach supports a framework for evaluating PCP function alongside genomics and *in silico* predictions toward understanding the pathophysiology impacts of genetic variations associated with human developmental disorders and gaining insight into Celsr1 function during development.

## Introduction

Tissue morphogenesis relies on the complex intersection of cell adhesion, polarity signaling and gene expression pathways (Saburi and McNeill, 2005; Heller and Fuchs, 2015; Shindo, 2018; Smith et al., 2022). Defects in cell-cell adhesion and polarity compromise tissue architecture, leading to a range of human developmental disorders resulting from impaired barrier function and aberrant signaling (McCrea et al., 2015; Garcia et al., 2018; Buckley and St Johnston, 2022). Cell surface adhesion molecules play major roles in facilitating cell movements during tissue development as well as in the establishment and maintenance of both apicobasal and planar polarity (Niessen et al., 2011; Niessen et al., 2012; Mui et al., 2016; Davey and Moens, 2017; Dzamba and DeSimone, 2018; Morgan et al., 2019). While apicobasal polarity is essential for the development of epithelial barrier properties (Niessen et al., 2012), planar cell polarity (PCP) is essential for orienting cells within the tissue plane to drive collective cell behaviors such as convergent extension during embryogenesis (Devenport, 2014; Butler and Wallingford, 2017; Shindo, 2018). Compromised PCP results in severe developmental defects across organ systems including heart and neural tube defects, ciliopathies and left/right patterning defects, cystic kidney disease and patterning defects of the inner ear and skin (Kibar et al., 2001; Curtin et al., 2003; Phillips et al., 2007; Devenport and Fuchs, 2008; Butler and Wallingford, 2017; Henderson et al., 2018; Shi, 2023).

The PCP pathway is conserved from flies to vertebrates and is characterized by a junctional complex comprised of a core set of multi-pass transmembrane proteins that asymmetrically assemble around a central adhesive plasma membrane domain (Devenport, 2014; Harrison et al., 2020). Frizzled (Fz in *Drosophila*) and Vangl (Vang in *Drosophila*) accumulate to opposite sides of cells along the anterior-posterior tissue axis, while atypical cadherin Celsr (Flamingo; Fmi in *Drosophila*) mediates homophilic cell-cell adhesion and thereby bridges the PCP junction across cells (Usui et al., 1999). In the mammalian skin, a key model system for studying vertebrate PCP, Celsr1 adhesion is essential for the accumulation of Fz6 and Vangl2 at cell borders (Devenport and Fuchs, 2008; Devenport et al., 2011; Stahley et al., 2021). Using the mouse mutant *Crsh*, harboring a single point mutation in the Celsr1 cadherin repeats, we demonstrated that lateral clustering, or *cis*-dimerization, is essential for both Celsr1 stability at the membrane and for organizing Fz6 and Vangl2 asymmetry at cell junctions (Stahley et al., 2021). Importantly, Celsr1 mutations in mouse models lead to developmental defects in the neural tube, heart, lung, kidney and skin, among others (Curtin et al., 2003; Yates et al., 2010; Murdoch et al., 2014; Henderson et al., 2018).

In humans, developmental birth defects occur in 3% of US newborns and are the leading cause of infant mortality in the US and Europe (Wallingford et al., 2013; Khokha et al., 2020). Neural tube defects (NTDs) and congenital heart defects (CHDs) are the most common, accounting for 0.1%–0.3% and 1% of live birth defects, respectively (Hoffman and Kaplan, 2002; Blencowe et al., 2018). Recent advances in genome sequencing have revealed mutations in nearly all PCP genes, highlighting the need to understand the mechanistic role of the PCP pathway in tissue patterning and how genetic defects contribute to human disease. Further, mutations in core PCP genes *CELSR1*, *FZD6*, and *VANGL2* are all associated with congenital birth defects, especially NTDs (Butler and Wallingford, 2017; Wang et al., 2019). However, it is not always clear if a specific genetic variation impacts protein function to contribute to a developmental defect or if it is simply a polymorphism that is harmless or even advantageous. Therefore, the development of screening approaches that can assess functional changes associated with genetic variations is essential.

Although shared *CELSR1* mutations among affected individuals are rare, *CELSR1* mutations occur with relatively high frequency in NTD and CHD patients, as high as 16% depending on the patient cohort (Allache et al., 2012; Qiao et al., 2016; Chen et al., 2018; Theis et al., 2022), highlighting the need to couple genetic screening with biological mechanism (Wang et al., 2019). Interestingly, many of these CELSR1 mutations map to the adhesive domain that engages in cell-cell adhesion and is essential for coordinating PCP. We sought to leverage these naturally occurring mutations associated with congenital birth defects to determine their impact on CELSR1 adhesion and PCP. Using a murine keratinocyte culture model system, we tested a cohort of 17 mutations mapping to the CELSR1/Celsr1 (mouse) cadherin repeats identified in CHD and NTD patients. We used multiple functional and structural assessments to understand how different disease-associated mutations perturb Celsr1 behavior at the cellular and molecular levels. Our findings reveal that numerous human disease-associated mutations in Celsr1 lead to alterations in Celsr1 function in adhesion, dynamics at the plasma membrane, and in the recruitment of other core PCP components to cell-cell junctions. Overall, our findings suggest that human disease-associated variants impact CELSR1 adhesion and support utilizing *in silico* pathogenicity predictions tools in combination with experimental validation to inform our understanding of CELSR1 function in development.

## Results

### Disease-associated Celsr1 variants exhibit differential junctional enrichment

The cadherin repeat-containing extracellular domain of Celsr1 is essential for Celsr1 enrichment at cell-cell junctions and for the co-recruitment of Fz6 and Vangl2 to coordinate PCP (Devenport and Fuchs, 2008). Mutation mapping studies of NTD and CHD patients have identified mutations in core PCP gene *CELSR1*, with many considered rare and predicted to be pathogenic (Allache et al., 2012; Robinson et al., 2012; Lei et al., 2014; Qiao et al., 2016; Chen et al., 2018; Wang et al., 2018). However, different *in silico* prediction tools were utilized. Further, a comprehensive functional assessment of these mutations is lacking. We hypothesized that mutations mapping to the CELSR1 cadherin domains contribute to human disease pathogenesis by disrupting Celsr1 function at cell junctions. From the aforementioned studies, we collated 17 *CELSR1* mutations that map to the extracellular cadherin (EC) repeat domains (Supplementary Table S1). The majority (14/17) were associated with NTD, while 2 were associated with CHD and 1 mutation was identified in separate patients from each disease cohort.

To assess potential functional impact of *CELSR1* single-nucleotide polymorphisms *in silico*, we evaluated all 17 cadherin-mapping CELSR1 mutations using the same set of sequence variant prediction tools. These tools included SIFT (Ng and Henikoff, 2001), integrative tools PolyPhen-2 (Adzhubei et al., 2010), FATHMM (Shihab et al., 2013; Shihab et al., 2014; Rogers et al., 2018), MutPred2 (Pejaver et al., 2020), CADD (Rentzsch et al., 2019) and Mutation Taster (Steinhaus et al., 2021), as well as AlphaMissense (Tordai et al., 2024), a newer, deep learning-based tool (Supplementary Table S2). In agreement with prior studies, these tools predicted a subset of mutations to be pathogenic but to varying degrees. For example, PolyPhen-2 and MutationTaster predicted 15/17 mutations to be pathogenic, only differing on two, while AlphaMissense predicted 4/17 to be pathogenic. Interestingly, V551M was the sole variant predicted to be pathogenic by all predictors, while in contrast, T1188S was the sole variant predicted to be non-pathogenic (Supplementary Table S2). Although these predictors offer potential insight, the disparities in predictive pathogenic impact of the mutations highlight a critical need for targeted functional assessments.

To complement the sequence variant tools and assess protein-level impacts, structural modeling with AlphaFold2 was utilized to render variant locations within the predicted cadherin domain structure followed by manual assessments of physiochemical characteristics and/or conformational changes introduced by the amino acid substitution (Supplementary Table S3). This analysis revealed many variants map near the inter-domain linker regions between EC domains where calcium ions bind, in particular R556W, R784W and P885L. To confirm this, available Protein Data Bank (PDB) structures from recent X-ray crystallography studies (Tamilselvan and Sotomayor, 2024) were utilized to measure the proximity to the nearest calcium ion for a given Celsr1 variant (Supplementary Figure S1; Supplementary Table S4). While we note the presence of a given mutation in available PDB structures including from a recent cryo-EM study (Bandekar et al., 2025), only those mapping to Celsr1 EC3-6 were able to be analyzed for calcium proximity. Of the 10 Celsr1 mutants mapping to residues present in recent resolved X-ray structures containing calcium, 6 were found to lie within 10 Å of a calcium ion and would be reasonably expected to impact calcium binding and inter-domain stability (Supplementary Table S4). Given the critical dependence of calcium binding on cadherin stability and cell-cell adhesion, we predict that residue proximity to calcium may shed light on possible pathogenicity mechanisms.

Stable enrichment of Celsr1 at adhesive cell-cell junctions is a requirement for functional PCP. Therefore, we expressed each disease-associated Celsr1 variant in keratinocytes and as carried out previously, measured accumulation at cell-cell interfaces, referred to as junctional enrichment, as a functional assessment of adhesion (Stahley et al., 2021; Basta et al., 2022). For these experiments, human equivalent mutations were introduced at the corresponding conserved residue in mouse Celsr1 (Figure 1A). Mouse Celsr1 was used to allow for direct comparison with the well-characterized PCP mouse mutant *Crash* (*Crsh*) (Curtin et al., 2003; Stahley et al., 2021), and to allow for subsequent analysis in a vertebrate developmental model system in future studies. Celsr1 variants were introduced into mouse keratinocytes and junctional enrichment at cell-cell borders was determined following a switch to medium containing high calcium to allow for junction assembly (Figure 1B). Wild type (WT) Celsr1 mediates cell-cell adhesion and robustly enriches at cell borders between neighboring keratinocytes (Devenport and Fuchs, 2008; Basta et al., 2022). By comparison, the PCP mutant Crsh, with characterized adhesion deficits, fails to mediate junctional enrichment (Devenport and Fuchs, 2008; Stahley et al., 2021). Of the 17 mutations tested, 13 displayed altered junctional enrichment relative to WT (Figure 1C). Among these, 12 mutants displayed reduced junctional enrichment, considered loss-of-function (LOF), while 1 mutant displayed increased junctional enrichment, considered gain-of-function (GOF) (Figure 1C). Variants in EC domains 3-9 exhibited reduced junctional enrichment or no change relative to WT, while a single GOF mutation was identified mapping to EC domain 6. Together, these results demonstrate that a majority of the known mutations in the Celsr1 EC domains associated with NTD/CHDs alter Celsr1 function.

**FIGURE 1:**
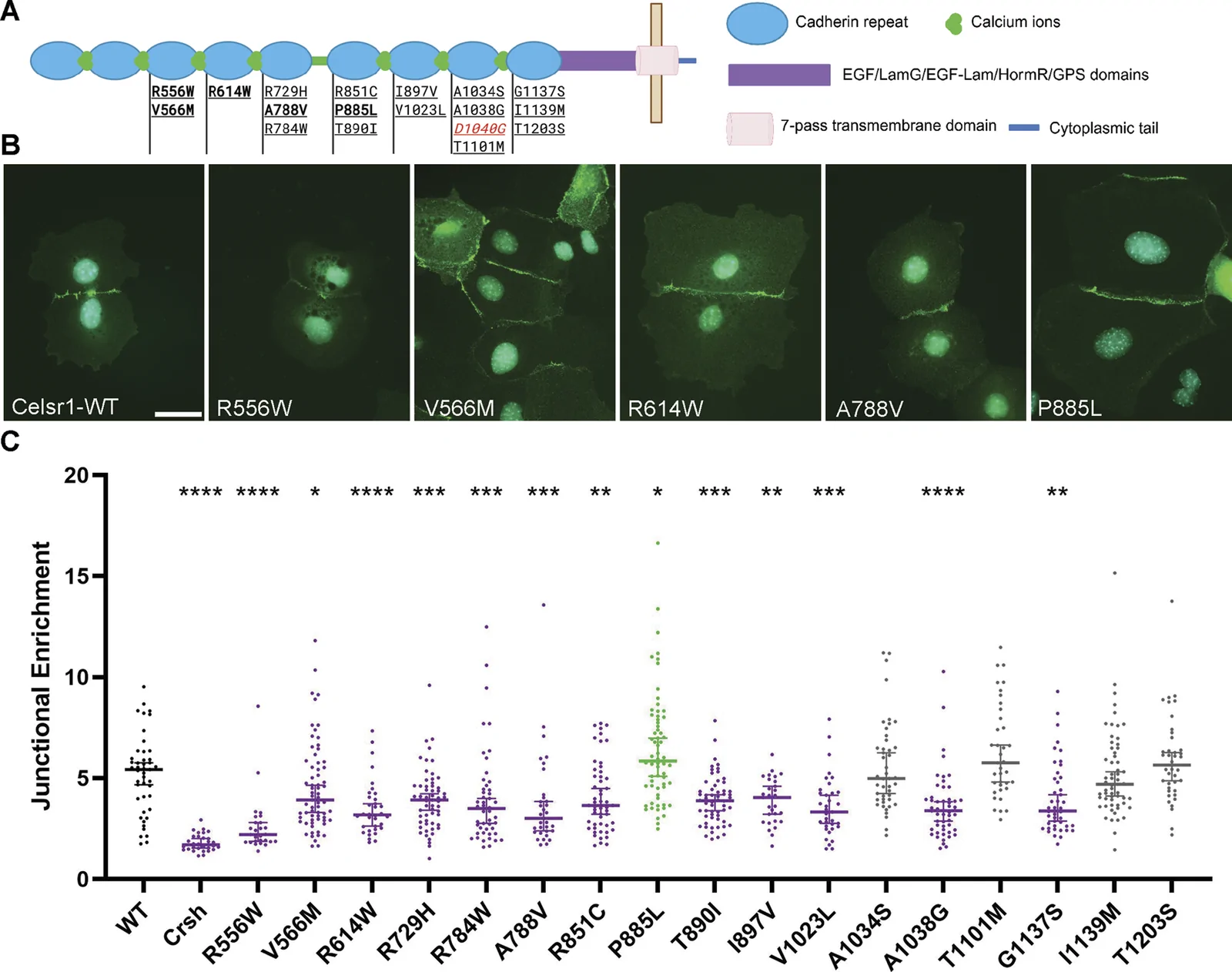
Celsr1 cadherin domain variants associated with human disease display differential junctional enrichment in keratinocytes. **(A)** Celsr1 schematic highlighting approximate location of corresponding mouse mutations within the cadherin repeats associated with human neural tube (NTD) and congenital heart defects (CHD). Protein domains are not drawn to scale. Schematic was created with BioRender. **(B)** GFP-tagged wild type (WT) Celsr1 and mutant variants expressed in cultured mouse keratinocytes. Cells were switched to high calcium medium for 24 h. Scale bar, 20 µm. **(C)** Quantification of Celsr1 junctional enrichment. Data pooled from 3-5 independent experiments per variant. Median ± 95% confidence interval. Outcome indications: black, WT; gray, statistically non-significant relative to WT; purple, decreased junctional enrichment; green, increased junctional enrichment. Mann-Whitney U test between variant and WT, *p < 0.05, **p < 0.01, ***p < 0.001, ****p < 0.0001; n = 27–67 junctions per variant.

We selected a subset of mutations for further analysis. R556W is the most N-terminal mutant in this analysis and located in EC3. Similar to Crsh, the R556W mutant exhibited LOF behavior with compromised junctional enrichment. In contrast, V566M exhibited only a slight reduction in junctional enrichment and was included for comparison to test for potential behaviors not fully revealed by the junctional enrichment assay. The R614W mutation occurs in EC4 and exhibits LOF behavior. A788V maps to EC5 and was previously identified as a trafficking mutant (Robinson et al., 2012). P885L exhibited GOF behavior with junctional enrichment that surpassed WT and thus was also included for subsequent analysis. Further, the human homolog of P885L, P870L, was previously identified as a GOF mutant in a Zebrafish model and was the only variant to be identified in both NTD and CHD patient cohorts (Qiao et al., 2016). Overall, these variants display comparable expression levels to WT, with only R556W and P885L displaying slightly higher and lower total expression respectively (Supplementary Figure S2A). Importantly, we find Celsr1 expression for a given cell-pair does not correlate with variant junctional enrichment (Supplementary Figures S2B,C). This variant subset was used to further assess the impact of disease-associated variants on Celsr1 function, as outlined below.

### Celsr1 mutants display differential stability at cell junctions

WT Celsr1 stably enriches at cell-cell borders and exhibits low fluorescence recovery after photobleaching (FRAP), indicating a very slow exchange of Celsr1 molecules that have engaged in cell-cell adhesion. Therefore, FRAP was utilized to examine mutational impact on Celsr1 mobility within the membrane (Figure 2A). As previously reported, WT displayed a low mobile fraction (16.5%). In contrast, higher mobile fractions were observed for mutants R614W (47.0%) and A788V (38.9%) (Figures 2B,C), consistent with their reduced junctional enrichment (Figures 1B,C; WT – 5.4, R614W – 3.2, A788V – 3.0). However, mutations R556W (19.0%) and V566M (21.1%) displayed minimal recovery and low mobile fractions similar to WT, inconsistent with their reduced junctional enrichment (Figures 1B,C; WT – 5.4, R556W – 2.2, V566M – 3.9). Lastly, the putative GOF mutant P885L, with the highest junctional enrichment ratio (Figure 1C), displayed reduced mobility within the plasma membrane with a mobile fraction of 10.2% (p = 0.065), though not to a degree that was statistically significant (Figures 2B,C). While these data modestly suggest that increased or decreased junctional enrichment in keratinocytes correlate with stability within the membrane, these data also indicate that these metrics are not always tightly coupled.

**FIGURE 2:**
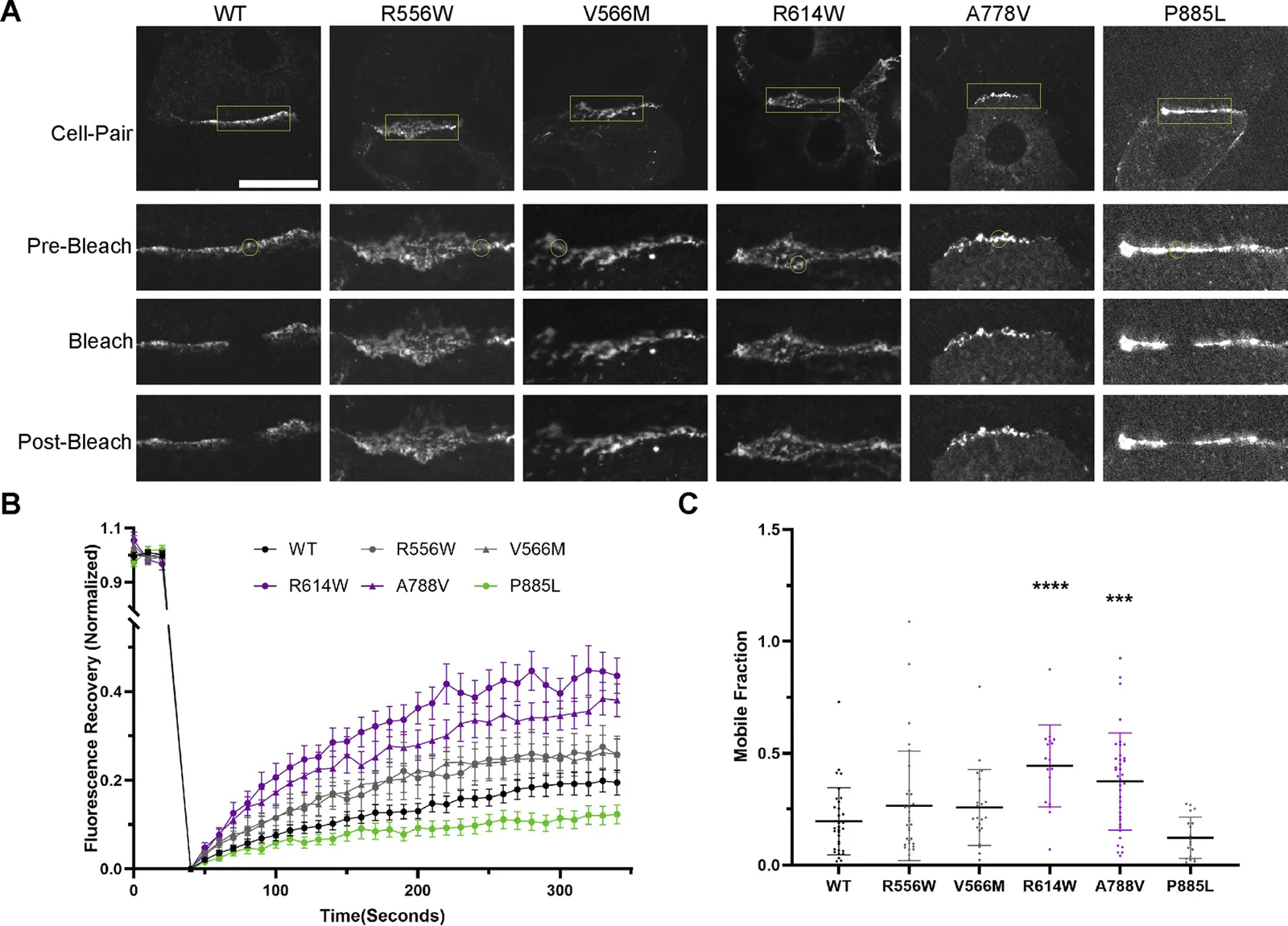
Fluorescence recovery after photobleaching (FRAP) reveals increased mobility for a portion of variants with decreased junctional enrichment. **(A)** Representative cell pairs of cultured keratinocytes transfected with the indicated Celsr1 variants and imaged by FRAP. Yellow circles indicate the photobleached region within the cell border of interest indicated by the yellow rectangles. Scale bar, 20 µm. **(B,C)** Normalized FRAP recovery curves **(B)** and mobile fraction quantification **(C)**. Outcome indications: black, WT; gray, statistically non-significant relative to WT; purple, increased recovery and mobile fraction; green, decreased recovery and mobile fraction. Data pooled from 3-6 independent experiments per variant. Error bars represent SEM **(B)**, median ± 95% confidence interval **(C)**. Mann-Whitney U test comparing variant mobile fraction to WT, ***p < 0.001, ****p < 0.0001; n = 15–38 junctions per variant, single ROI per junction.

### Celsr1 variants retain ability to interact with WT Celsr1

Previous studies indicate that Celsr1 engages in homotypic interactions in both *cis* (within the same cell) and *trans* (across cells) (Stahley et al., 2021). To assess *trans* interactions with WT, a mixed-cell assay was utilized in which keratinocytes expressing either mCherry-tagged WT (WT-mCh) or GFP-tagged variants were mixed and switched to high calcium medium to allow for junctional assembly (Figure 3A). Despite different junctional enrichments observed in Figure 1, all Celsr1 variants subsequently tested retained the ability to interact with WT in *trans* (Figure 3B), co-enriching with WT across cell-cell borders. Similarly, junctional enrichment of WT was unaffected by the presence of the Celsr1 variants (Figures 3B,D). Variant R556W displayed slightly reduced junctional enrichment at mixed-cell borders with WT (Figures 3B,C), suggesting diminished interactions in *trans*, though not to an appreciable degree to reach statistical significance.

**FIGURE 3:**
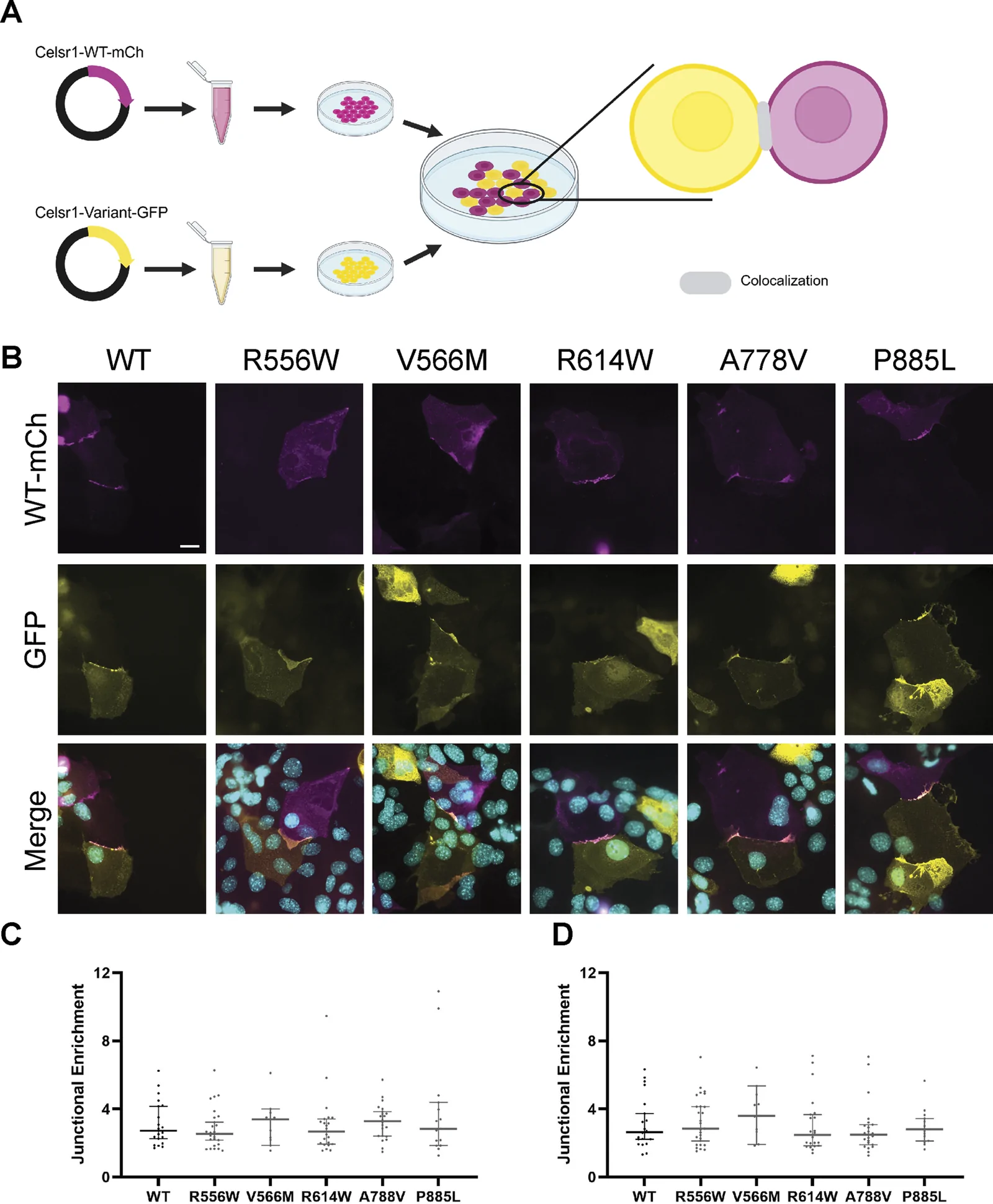
Celsr1 variants interact with WT in *trans.* **(A)** Schematic of mixed-cell junctional enrichment assay, created with BioRender. **(B)** Representative images of mixed-cell junctions between cells expressing either Celsr1-WT or the indicated mutant variant. Scale bar, 20 µm. **(C,D)** Mixed-cell junctional recruitment quantification. **(C)** Junctional enrichment of GFP-tagged variant indicated in mixed-cell junction with WT-mCh. **(D)** Junctional enrichment of WT-mCh in mixed-cell junction with indicated GFP-tagged variant. Outcome indications: black, WT; gray, statistically non-significant relative to WT. Data pooled from 2-3 independent experiments. Median ± 95% confidence interval. Mann-Whitney U test comparing junctional enrichment indicated to WT, all variants n.s.; n = 11–27 junctions per variant.

In addition to *trans* adhesive interactions across cells, lateral clustering of Celsr1 via *cis* interactions within the same cell are essential in forming stable Celsr1 puncta for functional PCP (Stahley et al., 2021). To determine how disease-associated mutations impact interactions with WT in *cis*, GFP-tagged Celsr1 variants were analyzed for the ability to enrich at cell junctions when co-expressed alongside WT Celsr1 in keratinocytes (Figure 4A). WT enrichment was unaffected by variant co-expression (Supplementary Figure S3). Surprisingly, we find all variants tested coenriched with WT (Figures 4B,C). This is in contrast to junctional enrichment for the LOF variants when solely expressed (Figures 1C, 4D). This finding suggests that the failure of LOF variants R556W, R614W and A788V to enrich at cell borders is rescued by the presence of WT Celsr1 within the same cell. Further, these findings suggest that *cis* interactions between variant and WT are unaffected in the mutations analyzed. Interestingly, GOF mutant P885L also displayed WT levels of enrichment when co-expressed with WT (Figure 4C).

**FIGURE 4:**
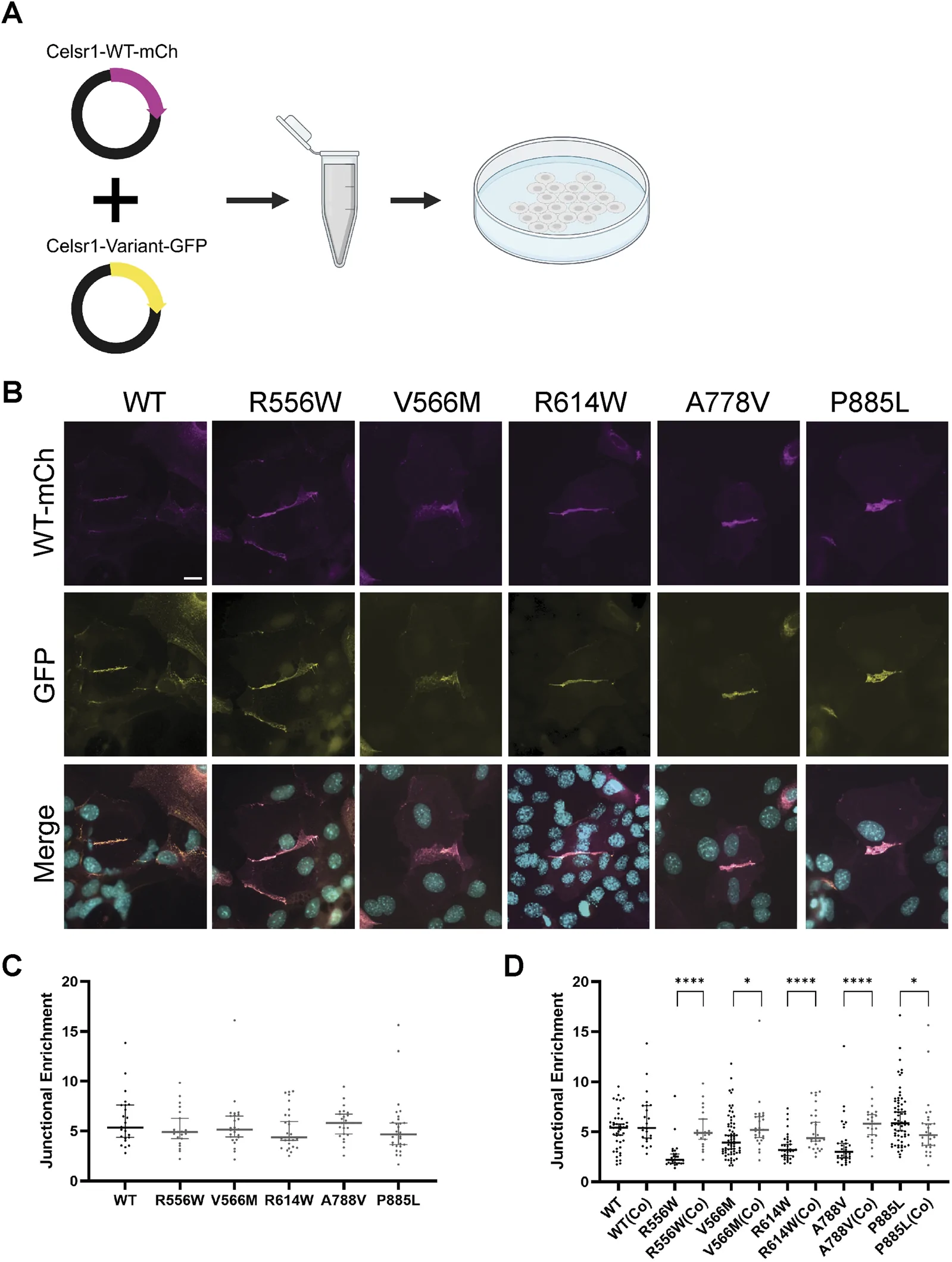
Celsr1 variants co-enrich with WT. **(A)** Schematic of co-transfection junctional enrichment assay, created with BioRender. **(B)** Representative images of mouse keratinocytes co-expressing Celsr1-WT along with the variants indicated. Scale bar, 20 µm. **(C,D)** Quantification of junctional enrichment. **(C)** Junctional enrichment of GFP-tagged variant indicated with WT-mCh co-expressed. **(D)** Junctional enrichment of variant indicated co-expressed with WT relative to variant alone (data sourced from experiments in Figure 1). Data pooled from 2-4 independent experiments. Median ± 95% confidence interval. Mann-Whitney U test relative to WT, *p < 0.05, ****p < 0.0001; n = 21–29 junctions per variant.

### Celsr1 cadherin domain variants impact Fz6 recruitment to cell borders

Beyond cell-cell adhesive interactions, Celsr1 is critical for the co-recruitment of core PCP transmembrane proteins Fz6 and Vangl2 at neighboring cell-cell interfaces. To determine mutational impact on PCP protein interactions, Celsr1 variants were co-expressed with either Fz6 or Vangl2, and assayed for their ability to co-recruit either Fz6 or Vangl2 to the cell borders (Figure 5). Except for GOF mutant P885L, all mutations examined failed to co-recruit Fz6 to cell borders, with Fz6 recruitment reduced compared to co-expression with WT (Figures 5A,C). The reduced junctional enrichment for R556W and R614W (Figure 1C) likely explains the inability to co-recruit Fz6 compared to WT and suggest that changes in Celsr1 *trans* adhesion that reduce junctional enrichment also impact Fz6 co-enrichment at cell junctions. Interestingly, variant V566M exhibits junctional enrichment comparable to WT but reduced Fz6 co-recruitment, suggesting that Celsr1-Fz6 interactions might be compromised by this mutation independently of Celsr1 homotypic adhesive interactions. In contrast, we find that Vangl2 co-enrichment was largely unaffected by the variants (Figure 5D).

**FIGURE 5:**
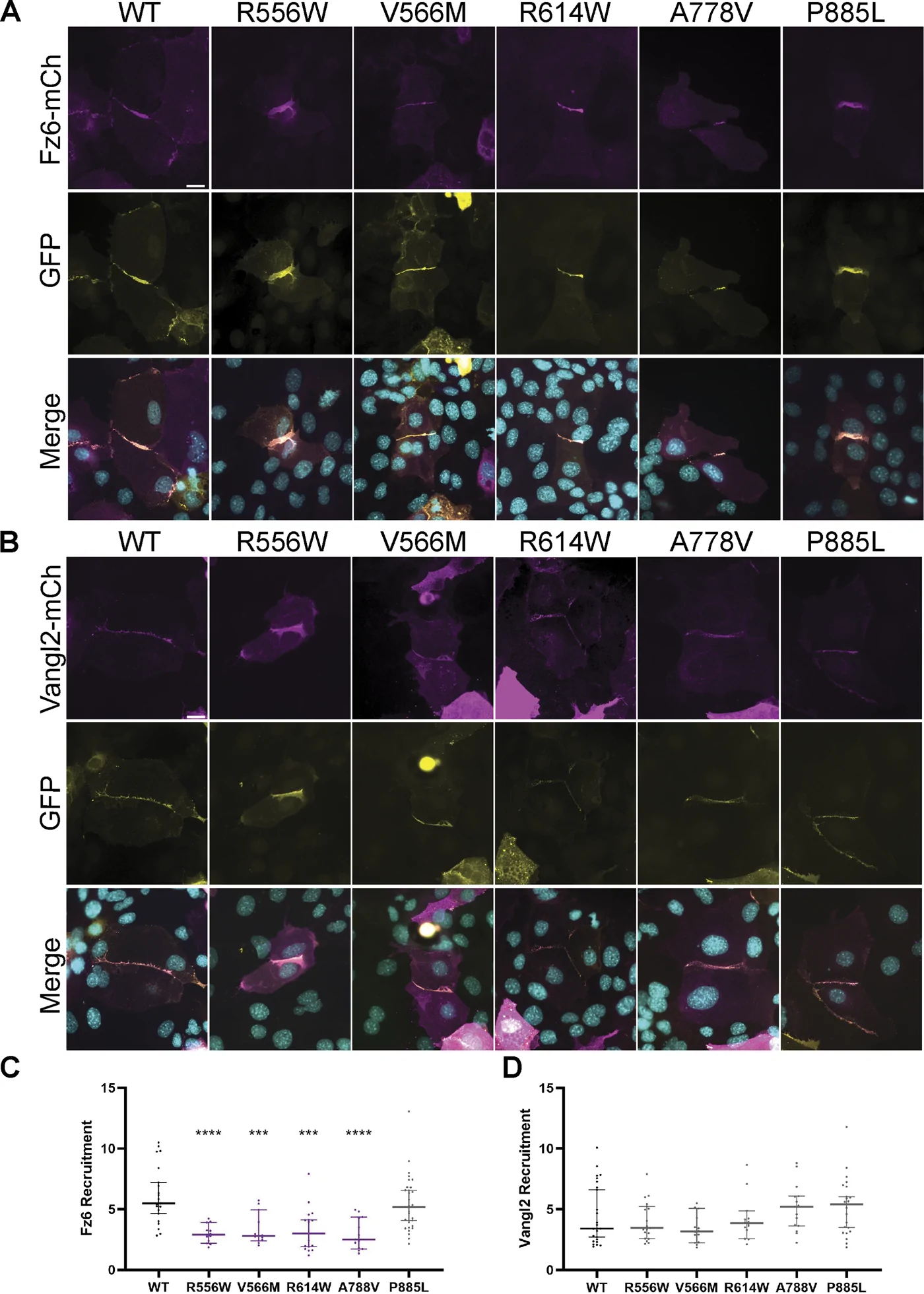
Celsr1 variants reduce Fz6 cell border recruitment. **(A)** Representative images of cultured keratinocytes co-expressing Celsr1-WT or variants indicated along with Fz6. Scale bar, 20 µm. **(B)** Representative images of cells co-expressing Celsr1-WT or variants indicated along with Vangl2. Scale bar, 20 µm. **(C,D)** Border enrichment quantification. **(C)** Fz6 recruitment measured by junctional enrichment when co-expressed with variants indicated. **(D)** Vangl2 recruitment measured by junctional enrichment when co-expressed with variants indicated. Data pooled from 2-4 independent experiments. Median ± 95% confidence interval. Mann-Whitney U test comparing Fz6 or Vangl2 recruitment when co-expressed with variant to WT control, ***p < 0.001, ****p < 0.0001, n = 13–31 junctions per variant with Fz6, n = 14–25 junctions per variant with Vangl2.

## Discussion

A major challenge in identifying causal variants in human disease is separating these from benign variations, modifiers or risk alleles, as only a small fraction of genetics variants cause developmental defects or diseases (Gonzaga-Jauregui et al., 2012; Marian, 2012). Functional assessments paired with *in silico* analysis provide a powerful approach to clarifying potential roles of sequence variations in developmental disorders. Here, utilizing a combination of *in silico* tools, structural modeling and *in vitro* functional analysis, we assess CELSR1/Celsr1 variants identified in patients with NTD/CHDs for differences that could underly human developmental defects and shed light on Celsr1-mediated coordination of PCP. With Celsr1 adhesion essential for PCP, we focus on mutations mapping to the CELSR1 cadherin repeats and analyze Celsr1 function using established assays for junctional enrichment, membrane dynamics and co-recruitment of other PCP proteins to cell-cell contacts. Our findings revealed both LOF and GOF variants and provide mechanistic insights into Celsr1 adhesion and how *CELSR1* mutations may contribute to human developmental defects.

Our initial screen revealed that the majority of Celsr1 mutations within the cadherin repeat region cause defects in the ability of Celsr1 to assemble and enrich at cell-cell interfaces. Our subsequent analysis focused on five mutants of interest, four LOF (R556W, V566M, R614W and A788V) and one GOF (P885L), with one of the LOF variants displaying reduced, yet most similar junctional enrichment to WT (V566M). All of these mutations lie within EC3-6, proximal to the proposed flexible partial calcium linker region between EC5 and EC6 (Jin et al., 2012) and N-terminally distal from the mouse mutations *Crsh* (EC8) and *Scy* (EC9) (Curtin et al., 2003). Recent structural studies have also implicated domains EC1-8 in the formation of the Celsr *trans* adhesive interface, as well as the importance of inter-domain calcium binding on Celsr1 confirmations and flexibility (Nishiguchi et al., 2023; Tamilselvan and Sotomayor, 2024; Bandekar et al., 2025). Unsurprisingly, our analysis of Celsr1 mutant proximity to the nearest calcium ion revealed that all mutants identified to be within 10 Å of a calcium ion were also found to display functional impacts on junctional enrichment. Thus, our functional analysis of these mutations is consistent with a key role for the Celsr1 cadherin repeats in cell adhesion and in the establishment of PCP complexes.

In addition to assessing the ability of each mutant to enrich at cell-cell junctions, we assayed the dynamics of select mutants. Celsr1 variants R614W and A788V, both identified as LOF (Figure 1) and located in EC4 and EC5 respectively, display higher mobile fractions after photobleaching. These results are consistent with previously characterized experimental mutations in Celsr1 and other cadherin family members that exhibit compromised adhesion (Harrison et al., 2011; Erami et al., 2015; Stahley et al., 2021). Interestingly, P885L, an apparent GOF variant was noticeably more stable than WT. These observations suggest that stability is essential for Celsr1 function in adhesion, but that dynamic properties of the cadherin are also essential for normal PCP function. It is tempting to speculate that the relative strength and/or stability of Celsr1 adhesive interactions are likely fine-tuned during development to meet tissue patterning requirements at different stages of embryogenesis.

Cadherins engage in both *cis* and *trans*-interactions that are essential for cell-cell adhesion and for transmission of forces across adhesive interfaces (Brasch et al., 2012; Mui et al., 2016). Adhesion forces generated from homotypic cadherin interactions have also been implicated as a key driver of convergent extension movements required for tissue morphogenesis (Niessen et al., 2011; Davey and Moens, 2017; Shindo, 2018). As Celsr1 and its *Drosophila* homolog Flamingo (Fmi) engage in homotypic *trans* interactions between cells, we assessed *trans* adhesive interactions between variants and WT Celsr1 by mixing cells expressing WT Celsr1 with keratinocytes expressing each of our Celsr1 mutants of interest. Surprisingly, no variants within the analyzed subset displayed reduced junctional enrichment in mixed-cell junctions with WT, nor were found to perturb that of co-expressed WT.

A hallmark of PCP cell junctions is the asymmetric organization of Fz6 and Vangl2 at opposing membranes of the Celsr1 cell-cell junction (Butler and Wallingford, 2017; Matsuda and Sokol, 2021). We observe that Fz6 co-recruitment at cell-cell borders is reduced when co-expressed with variants containing mutations in the proposed *trans* adhesive interface. These results suggest that Celsr1 adhesive defects associated with developmental disorders likely lead to PCP signaling deficiencies associated with reduced Fz6 recruitment. Interestingly, Vangl2 recruitment was not impacted by any of the variants tested. These observations are consistent with previous studies supporting extracellular domain interactions between Celsr3/Fmi and Fz and intracellular domain interactions between Fmi and Vang (Chen et al., 2008; Feng et al., 2021; Strutt et al., 2023). Additional studies are needed to map these interactions in finer detail and to understand how disease-associated mutations impact complex formation.

An important observation derived from our studies is that functional assessments of Celsr1 adhesion and membrane dynamics differed considerably from *in silico* pathogenicity scores (Table 1). Using a combined pathogenic hit score of 6/8 (ratio of *in silico* predictors classifying a given variant as pathogenic), we find 53% agreement between our *in vitro* outcomes and the *in silico* predictions. For those mutants nearest calcium, all with altered junctional enrichment, only half (3/6) were predicted pathogenic by the combined *in silico* predictors. Thus far, mutant proximity to calcium was the strongest predictor of *in vitro* functional impacts. Overall, these findings highlight the need to further develop and characterize Celsr1 adhesion and signaling assays to inform the significance of mutations identified in human disease, in alignment with recent functional studies evaluating *VANGL2* and *FZ6* mutations (Wang et al., 2019; Tian et al., 2020; Derrick et al., 2024). Since the initiation of the present study, additional CELSR1 variants mapping to the cadherin repeats have been identified: predicted damaging variant R823H in familial bicuspid aortic valve and variants A392V, R730W, D940N, R973Q and T1077M in hypoplastic left heart syndrome (Theis et al., 2022). Additional CELSR1 variants have also recently been linked to lymphedema, macrocephaly and seizures (Gonzalez-Garay et al., 2016; Sarasua et al., 2023; Bonardi et al., 2026). It will be important to understand the impact of these and other mutations on Celsr1 activity in functional assays and ultimately in vertebrate developmental models to understand how Celsr1 adhesion and PCP signaling contribute to normal human development.

**TABLE 1:** Comparison of combined *in silico* pathogenic predictions to experimental outcomes.

CELSR1 Cadherin Domain	Sequence variant		Combined *in silico* pathogenic prediction		Experimental outcomes	*In silico* vs. *in vitro* agreement?
	Human	Mouse	Hit score (%)	+ Damaging - Benign	Border enrichment (Previously reported)	
3	R541W	R556W	7/8 (88)	+	Decreased	Yes
3	V551M	V566M	8/8 (100)	+	Decreased	Yes
4	R599W	R614W	5/8 (63)	-	Decreased	No
5	R714H	R729H	4/8 (50)	-	Decreased	No
5	R769W	R784W	6/8 (75)	+	Decreased	Yes
5	A773V	A788V	3/8 (38)	-	Decreased (*LOF)	No
6	R836C	R851C	5/8 (63)	-	Decreased	No
6	P870L	P885L	6/8 (75)	+	Increased (**GOF)	Yes
6	T875I	T890I	5/8 (63)	-	Decreased	No
6	I882V	I897V	1/8 (13)	-	Decreased	No
8	V1008L	V1023L	6/8 (75)	+	Decreased	Yes
8	A1019S	A1034S	4/8 (50)	-	‘WT’	Yes
8	A1023G	A1038G	7/8 (88)	+	Decreased	Yes
8	T1086M	T1101M	6/8 (75)	+	‘WT’	No
9	G1122S	G1137S	5/8 (63)	-	Decreased	No
9	I1124M	I1139M	5/8 (63)	-	‘WT’	Yes
9	T1188S	T1203S	0/8 (0)	-	‘WT’	Yes
​						Agreement Score = 9/17 (53%)

* LOF, loss-of-function; reduced plasma membrane localization in MDCK cells (Robinson et al., 2012).

** GOF, gain-of-function; upregulation of both PCP and canonical WNT signaling in MDCK cells, induction on NTDs and CHDs in zebrafish (Qiao et al., 2016).

Hit score, ratio of *in silico* predictors classifying indicated variant as pathogenic (see Supplementary Table S2 for complete predictor outcomes). %, percentage representation of hit score. Combined pathogenic prediction, damaging (+) versus benign (−) if pathogenic hit score met a 75% threshold.

## Materials and methods

### Keratinocyte culture

Mouse keratinocytes (derived from P0 dorsal epidermis of *Mus musculus* CD-1 (Charles River, Strain #022) in the Devenport Lab), passage 20–33, were cultured at 37 °C with 5% CO_2_ in E-medium (3:1 DMEM/F12, US biological life science) with 10% fetal bovine serum (FBS, chelated) and 0.05 mM calcium (low calcium) supplemented with 400 ng/ml hydrocortisone, 180 μM adenine, 5 ng/ml EGF, 5 μg/ml insulin, 10 ng/ml cholera toxin.

### Molecular cloning and constructs

Mouse-equivalent Celsr1 mutations (Table 2) were introduced (VectorBuilder) into pEGFPN1-Celsr1-GFP (Devenport and Fuchs, 2008).

**TABLE 2:** Product information of key resources used in this study.

Reagent type or resource	Designation	Source or reference	Identifiers	Additional information
Antibody	Anti-GFP (Chicken polyclonal)	Abcam	Cat #ab13970	1:2000 (IF)
Antibody	Anti-RFP, also recognizes mCherry (Rabbit polyclonal)	Rockland Inc.	Cat #600–401–379	​
Antibody	Anti-Rabbit, Alexa Fluor 555 (Donkey)	Invitrogen	Cat #A-31572	​
Antibody	Anti-Chicken, Alexa Fluor 488 (Goat)	Abcam	Cat #ab150169	​
Antibody	Hoechst 33342	Invitrogen	Cat #H1399	​
Plasmid	pEGFPN1-Celsr1	Devenport and Fuchs (2008)	​	Full length WT Celsr1 with C-terminal GFP
Plasmid	pEGPFN1-Celsr1-mCherry	Stahley et al. (2021)	​	Full length WT Celsr1 with C-terminal mCherry
Plasmid	pEGFPN1-Celsr1-Crsh	Devenport and Fuchs (2008)	​	Celsr1 with D1040G mutation
Plasmid	pEGFPN1-Celsr1-R556W	This paper	VB210605-1067upk	Celsr1 with R556W mutation
Plasmid	pEGFPN1-Celsr1-V566M	This paper	VB210605-1074zwx	Celsr1 with V566M mutation
Plasmid	pEGFPN1-Celsr1-R614W	This paper	VB210605-1068ung	Celsr1 with R614W mutation
Plasmid	pEGFPN1-Celsr1-R729H	This paper	VB230123-1181qdf	Celsr1 with R729H mutation
Plasmid	pEGFPN1-Celsr1-R784W	This paper	VB210605-1070dtd	Celsr1 with R784W mutation
Plasmid	pEGFPN1-Celsr1-A788V	This paper	VB210605-1049unn	Celsr1 with A788V mutation
Plasmid	pEGFPN1-Celsr1-R851C	This paper	VB210605-1072hqe	Celsr1 with R851C mutation
Plasmid	pEGFPN1-Celsr1-P885L	This paper	VB210526-1329kqn	Celsr1 with P885L mutation
Plasmid	pEGFPN1-Celsr1-T891I	This paper	VB230123-1198dnt	Celsr1 with T891I mutation
Plasmid	pEGFPN1-Celsr1-I897V	This paper	VB210605-1060bpq	Celsr1 with I897V mutation
Plasmid	pEGFPN1-Celsr1-V1023L	This paper	VB210605-1077syc	Celsr1 with V1023L mutation
Plasmid	pEGFPN1-Celsr1-A1034S	This paper	VB230123-1156sna	Celsr1 with A1034S mutation
Plasmid	pEGFPN1-Celsr1-A1038G	This paper	VB210605-1051hab	Celsr1 with A1038G mutation
Plasmid	pEGFPN1-Celsr1-T1101M	This paper	VB230123-1199qnp	Celsr1 with T1101M mutation
Plasmid	pEGFPN1-Celsr1-G1137S	This paper	VB210605-1057acz	Celsr1 with G1137S mutation
Plasmid	pEGFPN1-Celsr1-I1139M	This paper	VB210605-1062mtp	Celsr1 with I1139M mutation
Plasmid	pEGFPN1-Celsr1-T1203S	This paper	VB210608-1055qxe	Celsr1 with T1203S mutation
Plasmid	pEGFPN1-Fz6-mCherry	Latario et al. (2026)	​	​
Plasmid	K14-Cherry-Vangl2	Devenport and Fuchs (2008)	​	​
Transfection reagent	Effectene	QIAGEN	Cat #301425	​
Software, algorithm	Fiji	https://fiji.sc	​	​
Software, algorithm	GraphPad Prism	http://www.graphpad.com	​	​
Software, algorithm	AlphaFold2	AlphaFold2.ipynb - Colab	​	​
Software, algorithm	SIFT	SIFT Single Protein Sequence	​	​
Software, algorithm	PolyPhen-2	PolyPhen-2: prediction of functional effects of human nsSNPs	​	​
Software, algorithm	FATHMM	fathmm - Home	​	​
Software, algorithm	CADD, v1.6	CADD - Combined Annotation Dependent Depletion	​	​
Software, algorithm	MutPred2	MutPred2: a predictor of impactful missense variants	​	​
Software, algorithm	MutationTaster 2025	MutationTaster - Study a chromosomal position	​	​
Software, algorithm	AlphaMissense	AlphaMissense	​	​

### Keratinocyte junctional enrichment, immunostaining and quantification

*Junctional enrichment and immunostaining.* Keratinocytes were cultured on fibronectin coated #1.5 glass coverslips for 24 h prior to transfection with Effectene (QIAGEN). Keratinocytes were switched to 1.5 mM calcium for 24 h to allow for junction formation prior to fixation in 4% paraformaldehyde for 10 min. After 3 PBS washes, cells were permeabilized in 0.1% Triton-X-100 (PBT) for 10 min with subsequent PBT washes. Cells were incubated with primary (in PBT) and secondary antibodies (see Table 2) for 1 h and 30 min respectively with PBT washes in between. Following a final PBS wash, coverslips were mounted on glass slides in ProLong^TM^ Diamond Antifade Mountant.

*Microscopy.* Widefield imaging was performed with either a Leica DMi8 microscope equipped with a HC PL APO CS2 60x/1.40 OIL objective or Zeiss Axio Observer.Z1 microscope equipped with an EC Plan-Neofluar 40x/1.30 Oil DIC M27 objective. All images for a given experiment were imaged with the same microscope and acquisition settings. Fiji was used for image analysis and subsequent quantification as described below.

*Quantification*. Keratinocyte junctional enrichment at cell-cell borders of neighboring cells is defined as the ratio of fluorescence intensity at cell borders relative to non-border regions. Using Fiji, a segmented line (width – 10) selection was drawn along the junction, non-junction regions within the cell pair, and a background region. Following background subtraction, junctional enrichment was calculated by dividing the border mean intensity by the average non-border mean intensity for the cell pair (or non-border mean intensity for the individual cell for mixed-cell assays). For co- and mixed-cell variations, see figure legends for additional sample and quantification details. A selection encompassing the periphery of the cell-pair was used for total mean intensity cell-pair quantification.

### Fluorescence recovery after photobleaching (FRAP)

Keratinocytes were seeded on fibronectin coated 8 well #1.5 glass bottom dishes from Cellvis (Cat # C8-1.5H-N). Following transfection and 24 h culturing in 1.5 mM calcium to induce junction formation, cells were imaged in phenol red free medium. FRAP experiments were performed on a Nikon Ti2-E equipped with a Yokogawa CSU-X1 spinning disk unit, LUNF XL laser unit, Semrock Di01-T405/488/568/647 polychroic mirror, a Chroma ET525/36m bandpass filter, Nikon Perfect Focus System, *Z* piezo stage, motorized *XY* stage, sCMOS camera (ORCA-Fusion BT, Hamamatsu Corp.) and a galvo miniscanner for selective photomanipulation and an Plan Apo TIRF 60x/1.49 NA oil immersion objective (Nikon). Cells were maintained at 5% CO_2_ and 37 °C. ROIs were drawn to encompass ∼2 µm of the cell border between adjacent cells. Images were then acquired every 10s with three pre-bleach timepoints until a recovery period of 5min. Normalized FRAP recovery curves were generated by calculating the normalized intensity as f: (F_t_–F_bleach_)/ (F_pre_–F_bleach_), where F_t_ represents the fluorescence intensity at times points during the acquisition. Mobile fractions were calculated as follows: M(f) = (F_end_–F_bleach_)/ (F_pre_–F_bleach_), where F_end_ represents the average of the final three timepoints. F_bleach_ represents the timepoint instantly after the photobleach, and F_pre_ represents the fluorescence intensity at the ROI before photobleaching.

### *In silico* modeling

The potential impact of missense mutations on Celsr1 function was evaluated using the following prediction tools: SIFT (Ng and Henikoff, 2001), PolyPhen-2 (Adzhubei et al., 2010), FATHMM (Shihab et al., 2013; Shihab et al., 2014; Rogers et al., 2018), CADD (Rentzsch et al., 2019) MutPred2 (Pejaver et al., 2020), MutationTaster (Steinhaus et al., 2021) and AlphaMissense (Tordai et al., 2024) as outlined in Table 3 below with human Celsr1 sequences and amino acid substitutions used as tool inputs.

**TABLE 3:** *In silico* predictors tools used in this study.

Tool	Input	Output	Table value
SIFT (Sorting Tolerant from Intolerant)	Uniprot fasta sequence: Q9NYQ6; list of amino acid substitutions	Score 0–1 ≤ 0.05 → deleterious > 0.05 → tolerated	Affected (++) Tolerated (−)
PolyPhen-2 (Polymorphism Phenotyping v2)	Ensembl ID: ENSG00000075275; Individual amino acid substitutions	Multiple sequence alignment and two scores, Hum Div (more sensitive model to detect rare, highly damaging mutations) and Hum Var (more conservative model to distinguish pathogenic vs. benign in human populations). For both models 0.0–0.15 → Benign 0.15–0.85 → Possibly Damaging 0.85–1.00 → Probably Damaging	Hum Var score: Probably damaging (++) Possibly damaging (+) Benign (−)
Inherited Disease FATHMM	Q9NYQ6; list of amino acid substitutions	Negative Values → Damaging Positive Values → Tolerated	Damaging (++) Tolerated (−)
FATHMM-XF (Enhanced accuracy in predicting the functional consequences of non-coding and coding single nucleotide variants)	GRCh37 chromosome position and substitution (Generated from MutationTaster 2021)	P-Value 0–1 <0.5 → deleterious >0.5 → neutral/ benign Extreme p-values indicate greatest accuracy	Pathogenic (++) Benign (−)
CADD (Combined Annotation Dependent Depletion), v1.6	GRCh37 chromosome position and substitution (Generated from MutationTaster 2021)	Scaled C-score 25–100 → Deleterious 20–25 → Possibly deleterious 0–20 → Tolerated	Deleterious (++) Possibly deleterious (+) Tolerated (−)
MutPred2	Uniprot fasta sequence: Q9NYQ6; list of amino acid substitutions	Score 0–1 0.0–0.49 → Benign 0.50–1.0 → Pathogenic	Pathogenic (++) Benign (−)
MutationTaster 2025	Celsr1 transcript ENST00000674500 (MANE); Individual nucleotide substitutions (HGMD)	Predictions (Deleterious or Benign) are based on random forest models. Tree vote depicts how many decision trees produced by the random forest model indicate deleterious predictions (before |) and how many decision trees indicate benign predictions (after |)	Deleterious (++) Benign (−)
AlphaMissense	Gene name: Celsr1, search for individual amino acid substitutions	Score 0–1 < 0.32 → Likely Benign 0.32–0.80 → Ambiguous >0.80 → Likely Pathogenic	Likely Pathogenic (++) Ambiguous (+) Likely Benign (−)

Celsr1 structure was modeled with Alphafold 2 (Jumper et al., 2021; Varadi et al., 2024). Due to the large size of Celsr1 (>3,000 amino acids), modeling was performed on portions of the cadherin repeat-containing extracellular region as follows: 1) the mouse equivalent of the human mutation was identified, 2) sequences for the mutation-containing cadherin repeat domain as well as the two adjacent protein domains were chosen for the query sequence (approximately 300–400 amino acids), 3) chosen sequence was run in ColabFold v1.5.5: AlphaFold2 using MMseqs2 (Jumper et al., 2021; Mirdita et al., 2022; Kim et al., 2025). The resulting protein structure model was visualized in ChimeraX to highlight the location of the mutated residue within the domain of interest (Meng et al., 2023). Potential disruption of post-translational modifications (PTMs) was determined using PhosphositePlus (Hornbeck et al., 2015). If a mutation had no predicted PTMs within its query sequence, then it was determined to result in no expected change to PTMs. Furthermore, mutations that did not fall within a predicted kinase recognition motif were determined to be unlikely to alter PTMs.

### Variant proximity to nearest calcium ion

Using PyMol (DeLano, 2002) and UniProt (UniProt, 2025) resources, PDB structures 8D40 and 7SZ8 (Tamilselvan and Sotomayor, 2024) were examined to measure the proximity of the nearest calcium ion in either structure to human equivalent residue to our mouse variants of interest. Enumerations of residues in both 8D40 and 7SZ8 are offset by 235 with the first residues of 8D40 and 7SZ8 corresponding to residues 236 and 558 in human CELSR1 respectively.

### Statistics

Statistical analysis was performed using Microsoft Excel, Prism (GraphPad Software, San Diego CA) or R. Non-parametric two-tailed Mann-Whitney U tests were used for comparisons due to non-normally distributed data. p-value significance defined as follows: p ≥ 0.05 (ns, not significant), *p < 0.05, **p < 0.01, ***p < 0.001, ****p < 0.0001. Refer to figure legends for additional statistical and experimental details.

## Data Availability

The authors will make available raw data generated and source files supporting this study without reservation.

## References

[B1] Adzhubei I. A. Schmidt S. Peshkin L. Ramensky V. E. Gerasimova A. Bork P. (2010). A method and server for predicting damaging missense mutations. Nat. Methods 7 (4), 248–249. 10.1038/nmeth0410-248 20354512 PMC2855889

[B2] Allache R. De Marco P. Merello E. Capra V. Kibar Z. (2012). Role of the planar cell polarity gene CELSR1 in neural tube defects and caudal agenesis. Birth Defects Res. A Clin. Mol. Teratol. 94 (3), 176–181. 10.1002/bdra.23002 22371354

[B3] Bandekar S. J. Garbett K. Kordon S. P. Dintzner E. E. Li J. Shearer T. (2025). Structural basis for regulation of CELSR1 by a compact module in its extracellular region. Nat. Commun. 16 (1), 3972. 10.1038/s41467-025-59319-8 40295529 PMC12038025

[B4] Basta L. P. Sil P. Jones R. A. Little K. A. Hayward-Lara G. Devenport D. (2022). Celsr1 and Celsr2 exhibit distinct adhesive interactions and contributions to planar cell polarity. Front. Cell Dev. Biol. 10, 1064907. 10.3389/fcell.2022.1064907 36712970 PMC9878842

[B5] Blencowe H. Kancherla V. Moorthie S. Darlison M. W. Modell B. (2018). Estimates of global and regional prevalence of neural tube defects for 2015: a systematic analysis. Ann. N. Y. Acad. Sci. 1414 (1), 31–46. 10.1111/nyas.13548 29363759

[B6] Bonardi C. M. Moller R. S. Ruiz-Reig N. Chai G. Madsen C. G. Bayat A. (2026). Biallelic variants in CELSR1 cause brain malformations, neurodevelopmental disorders and epilepsy in humans. Nat. Commun. 17, 862. 10.1038/s41467-025-67576-w 41530147 PMC12827284

[B7] Brasch J. Harrison O. J. Honig B. Shapiro L. (2012). Thinking outside the cell: how cadherins drive adhesion. Trends Cell Biol. 22 (6), 299–310. 10.1016/j.tcb.2012.03.004 22555008 PMC3385655

[B8] Buckley C. E. St Johnston D. (2022). Apical-basal polarity and the control of epithelial form and function. Nat. Rev. Mol. Cell Biol. 23 (8), 559–577. 10.1038/s41580-022-00465-y 35440694

[B9] Butler M. T. Wallingford J. B. (2017). Planar cell polarity in development and disease. Nat. Rev. Mol. Cell Biol. 18 (6), 375–388. 10.1038/nrm.2017.11 28293032 PMC5826606

[B10] Chen W. S. Antic D. Matis M. Logan C. Y. Povelones M. Anderson G. A. (2008). Asymmetric homotypic interactions of the atypical cadherin flamingo mediate intercellular polarity signaling. Cell 133 (6), 1093–1105. 10.1016/j.cell.2008.04.048 18555784 PMC2446404

[B11] Chen Z. Lei Y. Cao X. Zheng Y. Wang F. Bao Y. (2018). Genetic analysis of Wnt/PCP genes in neural tube defects. BMC Med. Genomics 11 (1), 38. 10.1186/s12920-018-0355-9 29618362 PMC5885375

[B12] Curtin J. A. Quint E. Tsipouri V. Arkell R. M. Cattanach B. Copp A. J. (2003). Mutation of Celsr1 disrupts planar polarity of inner ear hair cells and causes severe neural tube defects in the mouse. Curr. Biol. 13 (13), 1129–1133. 10.1016/s0960-9822(03)00374-9 12842012

[B13] Davey C. F. Moens C. B. (2017). Planar cell polarity in moving cells: think globally, act locally. Development 144 (2), 187–200. 10.1242/dev.122804 28096212 PMC5394761

[B14] DeLano W. L. (2002). The Pymol Molecular Graphics System. New York, USA: Schrodinger, LLC.

[B15] Derrick C. J. Szenker-Ravi E. Santos-Ledo A. Alqahtani A. Yusof A. Eley L. (2024). Functional analysis of germline VANGL2 variants using rescue assays of vangl2 knockout zebrafish. Hum. Mol. Genet. 33 (2), 150–169. 10.1093/hmg/ddad171 37815931 PMC10772043

[B16] Devenport D. (2014). The cell biology of planar cell polarity. J. Cell Biol. 207 (2), 171–179. 10.1083/jcb.201408039 25349257 PMC4210441

[B17] Devenport D. Fuchs E. (2008). Planar polarization in embryonic epidermis orchestrates global asymmetric morphogenesis of hair follicles. Nat. Cell Biol. 10 (11), 1257–1268. 10.1038/ncb1784 18849982 PMC2607065

[B18] Devenport D. Oristian D. Heller E. Fuchs E. (2011). Mitotic internalization of planar cell polarity proteins preserves tissue polarity. Nat. Cell Biol. 13 (8), 893–902. 10.1038/ncb2284 21743464 PMC3149741

[B19] Dzamba B. J. DeSimone D. W. (2018). Extracellular matrix (ECM) and the sculpting of embryonic tissues. Curr. Top. Dev. Biol. 130, 245–274. 10.1016/bs.ctdb.2018.03.006 29853179

[B20] Erami Z. Timpson P. Yao W. Zaidel-Bar R. Anderson K. I. (2015). There are four dynamically and functionally distinct populations of E-cadherin in cell junctions. Biol. Open 4 (11), 1481–1489. 10.1242/bio.014159 26471767 PMC4728362

[B21] Feng B. Freitas A. E. Gorodetski L. Wang J. Tian R. Lee Y. R. (2021). Planar cell polarity signaling components are a direct target of beta-amyloid-associated degeneration of glutamatergic synapses. Sci. Adv. 7 (34), eabh2307. 10.1126/sciadv.abh2307 34407949 PMC8373119

[B22] Garcia M. A. Nelson W. J. Chavez N. (2018). Cell-Cell junctions organize structural and signaling networks. Cold Spring Harb. Perspect. Biol. 10 (4). 10.1101/cshperspect.a029181 28600395 PMC5773398

[B23] Gonzaga-Jauregui C. Lupski J. R. Gibbs R. A. (2012). Human genome sequencing in health and disease. Annu. Rev. Med. 63, 35–61. 10.1146/annurev-med-051010-162644 22248320 PMC3656720

[B24] Gonzalez-Garay M. L. Aldrich M. B. Rasmussen J. C. Guilliod R. Lapinski P. E. King P. D. (2016). A novel mutation in CELSR1 is associated with hereditary lymphedema. Vasc. Cell 8, 1. 10.1186/s13221-016-0035-5 26855770 PMC4743364

[B25] Harrison O. J. Jin X. Hong S. Bahna F. Ahlsen G. Brasch J. (2011). The extracellular architecture of adherens junctions revealed by crystal structures of type I cadherins. Structure 19 (2), 244–256. 10.1016/j.str.2010.11.016 21300292 PMC3070544

[B26] Harrison C. Shao H. Strutt H. Strutt D. (2020). Molecular mechanisms mediating asymmetric subcellular localisation of the core planar polarity pathway proteins. Biochem. Soc. Trans. 48 (4), 1297–1308. 10.1042/BST20190404 32820799 PMC7458395

[B27] Heller E. Fuchs E. (2015). Tissue patterning and cellular mechanics. J. Cell Biol. 211 (2), 219–231. 10.1083/jcb.201506106 26504164 PMC4621832

[B28] Henderson D. J. Long D. A. Dean C. H. (2018). Planar cell polarity in organ formation. Curr. Opin. Cell Biol. 55, 96–103. 10.1016/j.ceb.2018.06.011 30015152

[B29] Hoffman J. I. Kaplan S. (2002). The incidence of congenital heart disease. J. Am. Coll. Cardiol. 39 (12), 1890–1900. 10.1016/s0735-1097(02)01886-7 12084585

[B30] Hornbeck P. V. Zhang B. Murray B. Kornhauser J. M. Latham V. Skrzypek E. (2015). PhosphoSitePlus, 2014: mutations, PTMs and recalibrations. Nucleic Acids Res. 43 (Database issue), D512–D520. 10.1093/nar/gku1267 25514926 PMC4383998

[B31] Jin X. Walker M. A. Felsovalyi K. Vendome J. Bahna F. Mannepalli S. (2012). Crystal structures of drosophila N-cadherin ectodomain regions reveal a widely used class of Ca(2)+-free interdomain linkers. Proc. Natl. Acad. Sci. U. S. A. 109 (3), E127–E134. 10.1073/pnas.1117538108 22171007 PMC3271863

[B32] Jumper J. Evans R. Pritzel A. Green T. Figurnov M. Ronneberger O. (2021). Highly accurate protein structure prediction with AlphaFold. Nature 596 (7873), 583–589. 10.1038/s41586-021-03819-2 34265844 PMC8371605

[B33] Khokha M. K. Liu K. J. Wallingford J. B. (2020). Challenges and opportunities at the interface of birth defects, human genetics and developmental biology. Development 147 (21). 10.1242/dev.197871 33168578 PMC8316676

[B34] Kibar Z. Vogan K. J. Groulx N. Justice M. J. Underhill D. A. Gros P. (2001). Ltap, a mammalian homolog of drosophila strabismus/van gogh, is altered in the mouse neural tube mutant Loop-tail. Nat. Genet. 28 (3), 251–255. 10.1038/90081 11431695

[B35] Kim G. Lee S. Levy Karin E. Kim H. Moriwaki Y. Ovchinnikov S. (2025). Easy and accurate protein structure prediction using ColabFold. Nat. Protoc. 20 (3), 620–642. 10.1038/s41596-024-01060-5 39402428

[B76] Latario S. G. Saba T. Trejo B. Giang W. Zimmer S. E. Devenport D. (2026). Planar cell polarity complex internalization is dependent on Celsr1 cis-dimerization. iScience 29 (8), 116761. 10.1016/j.isci.2026.116761 42502379 PMC13400783

[B36] Lei Y. Zhu H. Yang W. Ross M. E. Shaw G. M. Finnell R. H. (2014). Identification of novel CELSR1 mutations in spina bifida. PLoS One 9 (3), e92207. 10.1371/journal.pone.0092207 24632739 PMC3954890

[B37] Marian A. J. (2012). Challenges in medical applications of whole exome/genome sequencing discoveries. Trends Cardiovasc Med. 22 (8), 219–223. 10.1016/j.tcm.2012.08.001 22921985 PMC3496831

[B38] Matsuda M. Sokol S. Y. (2021). Xenopus neural tube closure: a vertebrate model linking planar cell polarity to actomyosin contractions. Curr. Top. Dev. Biol. 145, 41–60. 10.1016/bs.ctdb.2021.04.001 34074535

[B39] McCrea P. D. Maher M. T. Gottardi C. J. (2015). Nuclear signaling from cadherin adhesion complexes. Curr. Top. Dev. Biol. 112, 129–196. 10.1016/bs.ctdb.2014.11.018 25733140 PMC4410048

[B40] Meng E. C. Goddard T. D. Pettersen E. F. Couch G. S. Pearson Z. J. Morris J. H. (2023). UCSF ChimeraX: tools for structure building and analysis. Protein Sci. 32 (11), e4792. 10.1002/pro.4792 37774136 PMC10588335

[B41] Mirdita M. Schutze K. Moriwaki Y. Heo L. Ovchinnikov S. Steinegger M. (2022). ColabFold: making protein folding accessible to all. Nat. Methods 19 (6), 679–682. 10.1038/s41592-022-01488-1 35637307 PMC9184281

[B42] Morgan R. K. Anderson G. R. Arac D. Aust G. Balenga N. Boucard A. (2019). The expanding functional roles and signaling mechanisms of adhesion G protein-coupled receptors. Ann. N. Y. Acad. Sci. 1456 (1), 5–25. 10.1111/nyas.14094 31168816 PMC7891679

[B43] Mui K. L. Chen C. S. Assoian R. K. (2016). The mechanical regulation of integrin-cadherin crosstalk organizes cells, signaling and forces. J. Cell Sci. 129 (6), 1093–1100. 10.1242/jcs.183699 26919980 PMC4813297

[B44] Murdoch J. N. Damrau C. Paudyal A. Bogani D. Wells S. Greene N. D. (2014). Genetic interactions between planar cell polarity genes cause diverse neural tube defects in mice. Dis. Model Mech. 7 (10), 1153–1163. 10.1242/dmm.016758 25128525 PMC4174526

[B45] Ng P. C. Henikoff S. (2001). Predicting deleterious amino acid substitutions. Genome Res. 11 (5), 863–874. 10.1101/gr.176601 11337480 PMC311071

[B46] Niessen C. M. Leckband D. Yap A. S. (2011). Tissue organization by cadherin adhesion molecules: dynamic molecular and cellular mechanisms of morphogenetic regulation. Physiol. Rev. 91 (2), 691–731. 10.1152/physrev.00004.2010 21527735 PMC3556819

[B47] Niessen M. T. Iden S. Niessen C. M. (2012). The *in vivo* function of mammalian cell and tissue polarity regulators-how to shape and maintain the epidermal barrier. J. Cell Sci. 125 (15), 3501–3510. 10.1242/jcs.092890 22935653

[B48] Nishiguchi S. Kasai R. S. Uchihashi T. (2023). Antiparallel dimer structure of CELSR cadherin in solution revealed by high-speed atomic force microscopy. Proc. Natl. Acad. Sci. U. S. A. 120 (18), e2302047120. 10.1073/pnas.2302047120 37094146 PMC10160967

[B49] Pejaver V. Urresti J. Lugo-Martinez J. Pagel K. A. Lin G. N. Nam H. J. (2020). Inferring the molecular and phenotypic impact of amino acid variants with MutPred2. Nat. Commun. 11 (1), 5918. 10.1038/s41467-020-19669-x 33219223 PMC7680112

[B50] Phillips H. M. Rhee H. J. Murdoch J. N. Hildreth V. Peat J. D. Anderson R. H. (2007). Disruption of planar cell polarity signaling results in congenital heart defects and cardiomyopathy attributable to early cardiomyocyte disorganization. Circ. Res. 101 (2), 137–145. 10.1161/CIRCRESAHA.106.142406 17556662

[B51] Qiao X. Liu Y. Li P. Chen Z. Li H. Yang X. (2016). Genetic analysis of rare coding mutations of CELSR1-3 in congenital heart and neural tube defects in Chinese people. Clin. Sci. (Lond) 130 (24), 2329–2340. 10.1042/CS20160686 27756857

[B52] Rentzsch P. Witten D. Cooper G. M. Shendure J. Kircher M. (2019). CADD: predicting the deleteriousness of variants throughout the human genome. Nucleic Acids Res. 47 (1), D886–D894. 10.1093/nar/gky1016 30371827 PMC6323892

[B53] Robinson A. Escuin S. Doudney K. Vekemans M. Stevenson R. E. Greene N. D. (2012). Mutations in the planar cell polarity genes CELSR1 and SCRIB are associated with the severe neural tube defect craniorachischisis. Hum. Mutat. 33 (2), 440–447. 10.1002/humu.21662 22095531 PMC4772123

[B54] Rogers M. F. Shihab H. A. Mort M. Cooper D. N. Gaunt T. R. Campbell C. (2018). FATHMM-XF: accurate prediction of pathogenic point mutations via extended features. Bioinformatics 34 (3), 511–513. 10.1093/bioinformatics/btx536 28968714 PMC5860356

[B55] Saburi S. McNeill H. (2005). Organising cells into tissues: new roles for cell adhesion molecules in planar cell polarity. Curr. Opin. Cell Biol. 17 (5), 482–488. 10.1016/j.ceb.2005.08.011 16099635

[B56] Sarasua S. M. DeLuca J. M. Rogers C. Phelan K. Rennert L. Powder K. E. (2023). Head size in Phelan-McDermid Syndrome: a literature review and pooled analysis of 198 patients identifies candidate genes on 22q13. Genes (Basel) 14 (3), 540. 10.3390/genes14030540 36980813 PMC10048319

[B57] Shi D. L. (2023). Planar cell polarity regulators in asymmetric organogenesis during development and disease. J. Genet. Genomics 50 (2), 63–76. 10.1016/j.jgg.2022.06.007 35809777

[B58] Shihab H. A. Gough J. Cooper D. N. Day I. N. Gaunt T. R. (2013). Predicting the functional consequences of cancer-associated amino acid substitutions. Bioinformatics 29 (12), 1504–1510. 10.1093/bioinformatics/btt182 23620363 PMC3673218

[B59] Shihab H. A. Gough J. Mort M. Cooper D. N. Day I. N. Gaunt T. R. (2014). Ranking non-synonymous single nucleotide polymorphisms based on disease concepts. Hum. Genomics 8 (1), 11. 10.1186/1479-7364-8-11 24980617 PMC4083756

[B60] Shindo A. (2018). Models of convergent extension during morphogenesis. Wiley Interdiscip. Rev. Dev. Biol. 7 (1). 10.1002/wdev.293 28906063 PMC5763355

[B61] Smith S. J. Guillon E. Holley S. A. (2022). The roles of inter-tissue adhesion in development and morphological evolution. J. Cell Sci. 135 (9), jcs259579. 10.1242/jcs.259579 35522159 PMC9264361

[B62] Stahley S. N. Basta L. P. Sharan R. Devenport D. (2021). Celsr1 adhesive interactions mediate the asymmetric organization of planar polarity complexes. Elife 10, e62097. 10.7554/eLife.62097 33529151 PMC7857726

[B63] Steinhaus R. Proft S. Schuelke M. Cooper D. N. Schwarz J. M. Seelow D. (2021). MutationTaster2021. Nucleic Acids Res. 49 (W1), W446–W451. 10.1093/nar/gkab266 33893808 PMC8262698

[B64] Strutt H. Warrington S. Madathil A. C. K. Langenhan T. Strutt D. (2023). Molecular symmetry breaking in the Frizzled-dependent planar polarity pathway. Curr. Biol. 33 (24), 5340–5354 e5346. 10.1016/j.cub.2023.10.071 37995695 PMC7616066

[B65] Tamilselvan E. Sotomayor M. (2024). CELSR1, a core planar cell polarity protein, features a weakly adhesive and flexible cadherin ectodomain. Structure 32 (4), 476–491. 10.1016/j.str.2024.01.003 38307021

[B66] Theis J. L. Niaz T. Sundsbak R. S. Fogarty Z. C. Bamlet W. R. Hagler D. J. (2022). CELSR1 risk alleles in familial bicuspid aortic valve and hypoplastic left heart syndrome. Circ. Genom Precis. Med. 15 (2), e003523. 10.1161/CIRCGEN.121.003523 35133174

[B67] Tian T. Lei Y. Chen Y. Karki M. Jin L. Finnell R. H. (2020). Somatic mutations in planar cell polarity genes in neural tissue from human fetuses with neural tube defects. Hum. Genet. 139 (10), 1299–1314. 10.1007/s00439-020-02172-0 32356230 PMC7487040

[B68] Tordai H. Torres O. Csepi M. Padanyi R. Lukacs G. L. Hegedus T. (2024). Analysis of AlphaMissense data in different protein groups and structural context. Sci. Data 11 (1), 495. 10.1038/s41597-024-03327-8 38744964 PMC11094042

[B69] UniProt C. (2025). UniProt: the protein knowledgebase in 2025. Nucleic Acids Res. 53 (D1), D609–D617. 10.1093/nar/gkae1010 39552041 PMC11701636

[B70] Usui T. Shima Y. Shimada Y. Hirano S. Burgess R. W. Schwarz T. L. (1999). Flamingo, a seven-pass transmembrane cadherin, regulates planar cell polarity under the control of frizzled. Cell 98 (5), 585–595. 10.1016/s0092-8674(00)80046-x 10490098

[B71] Varadi M. Bertoni D. Magana P. Paramval U. Pidruchna I. Radhakrishnan M. (2024). AlphaFold protein structure database in 2024: providing structure coverage for over 214 million protein sequences. Nucleic Acids Res. 52 (D1), D368–D375. 10.1093/nar/gkad1011 37933859 PMC10767828

[B72] Wallingford J. B. Niswander L. A. Shaw G. M. Finnell R. H. (2013). The continuing challenge of understanding, preventing, and treating neural tube defects. Science 339 (6123), 1222002. 10.1126/science.1222002 23449594 PMC3677196

[B73] Wang L. Xiao Y. Tian T. Jin L. Lei Y. Finnell R. H. (2018). Digenic variants of planar cell polarity genes in human neural tube defect patients. Mol. Genet. Metab. 124 (1), 94–100. 10.1016/j.ymgme.2018.03.005 29573971 PMC5966321

[B74] Wang M. Marco P. Capra V. Kibar Z. (2019). Update on the role of the non-canonical wnt/planar cell polarity pathway in neural tube defects. Cells 8 (10). 10.3390/cells8101198 31590237 PMC6829399

[B75] Yates L. L. Schnatwinkel C. Murdoch J. N. Bogani D. Formstone C. J. Townsend S. (2010). The PCP genes Celsr1 and Vangl2 are required for normal lung branching morphogenesis. Hum. Mol. Genet. 19 (11), 2251–2267. 10.1093/hmg/ddq104 20223754 PMC2865378

