# Supplementary material for: Functional assessment of CELSR1 variants associated with human developmental defects
Source: Front Cell Dev Biol. 2026 Oct 8;14:1818587. doi: 10.3389/fcell.2026.1818587 (PMC13687084; doi:10.3389/fcell.2026.1818587)
Supplement: Supplementary file 1 [file Supplementaryfile1.pdf]

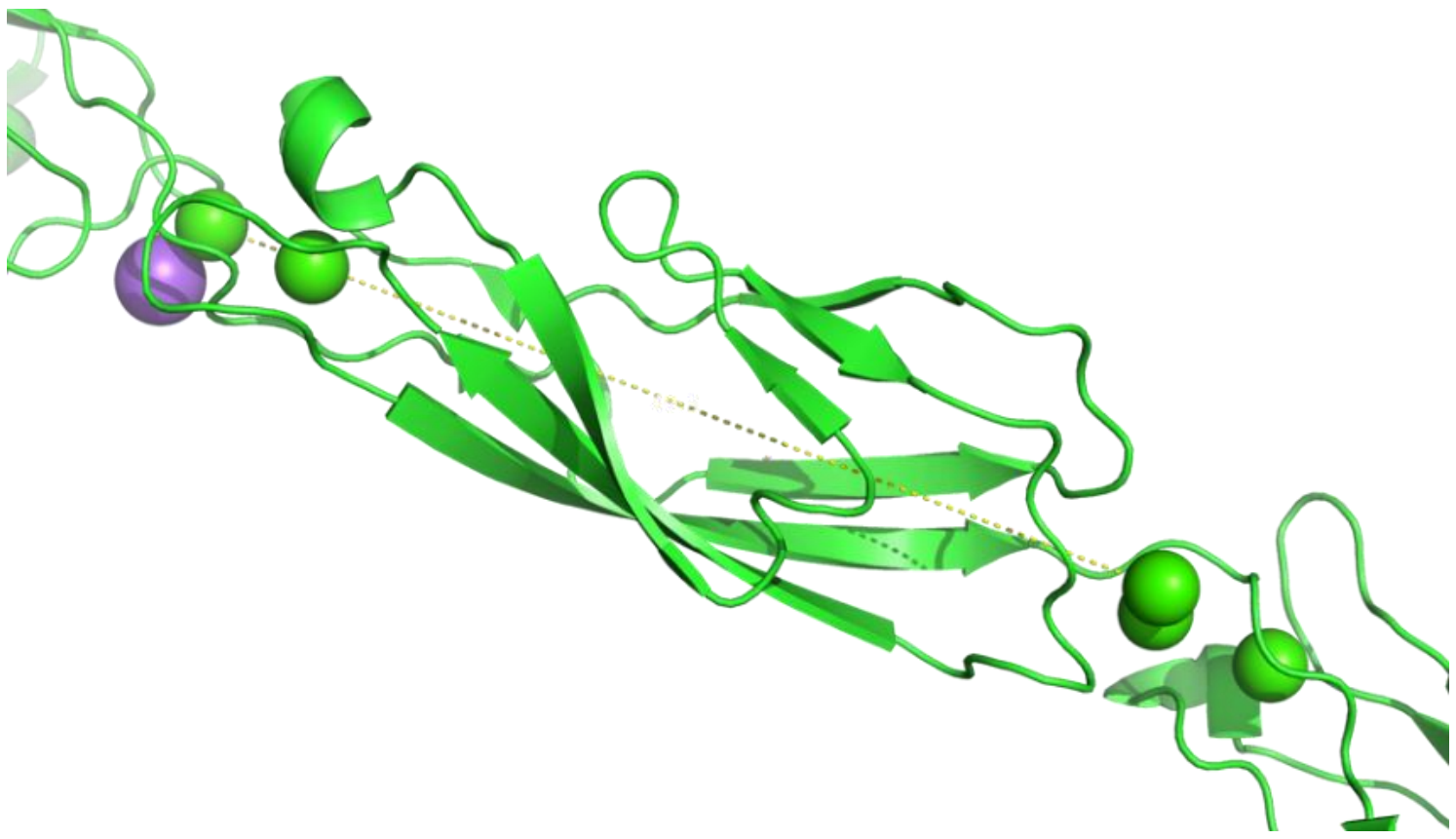

SUPPLEMENTARY FIGURE S1

Inter-calcium distance in 8D40 for EC2 domain. Dashed yellow line = 49.3 angstroms.

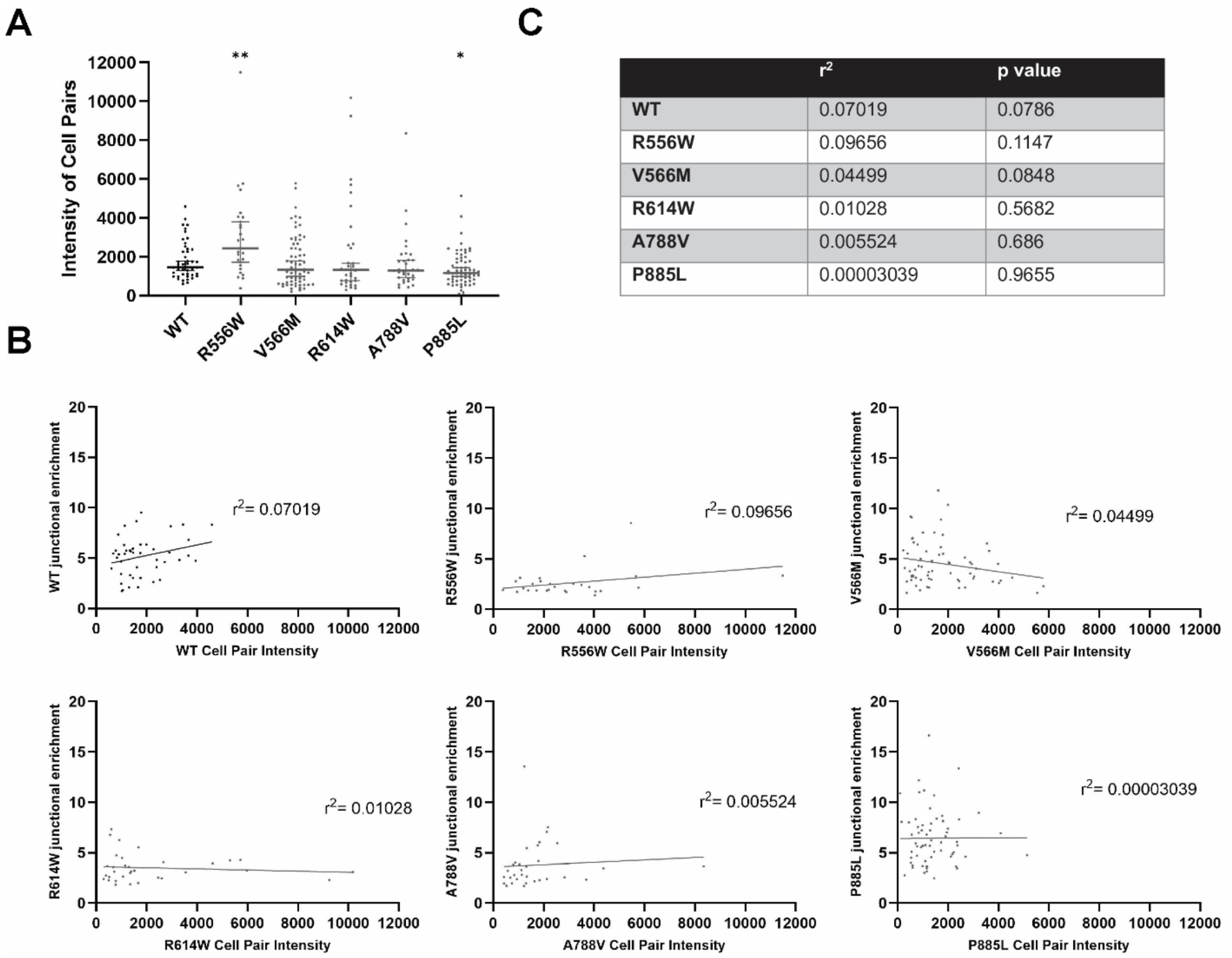

	r^2	p value
WT	0.07019	0.0786
R556W	0.09656	0.1147
V566M	0.04499	0.0848
R614W	0.01028	0.5682
A788V	0.005524	0.686
P885L	0.00003039	0.9655

SUPPLEMENTARY FIGURE S2

Celsr1 junctional enrichment does not correlate with differences in Celsr1 expression. (**A**) Quantification of total mean intensity of cell pairs expressing the GFP-tagged Celsr1 variants indicated. Data pooled from 3-5 independent experiments per variant. Median ± 95% confidence interval. Two-tailed Mann-Whitney U test between variant and WT expression, *p < 0.05, **p < 0.01; n = 27-67 junctions per variant. **(B)** Plot of Celsr1-GFP expression (total mean intensity from plots in panel A) of an individual cell pair relative to its corresponding junctional enrichment score (Figure 1C). **(C)** Corresponding r^2 and p values for plots in panel B.

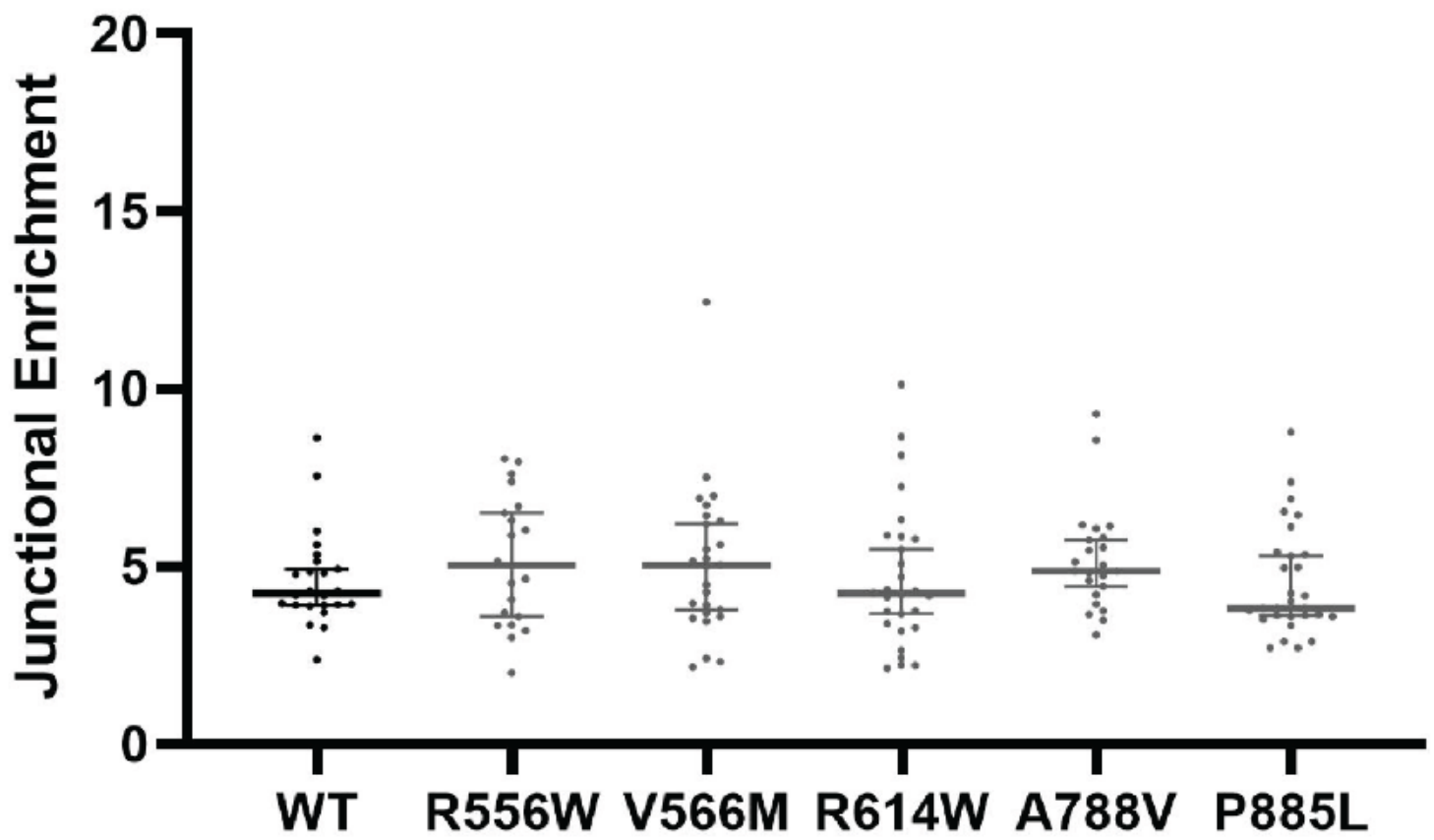

SUPPLEMENTARY FIGURE S3

Celsr1-WT junctional enrichment is unaffected by co-expression with disease-associated variants. **(A)** Junctional enrichment of mCherry-tagged WT when co-expressed with GFP-tagged WT or variants indicated. Median ± 95% confidence interval. Co-transfection data pooled from 2-4 independent experiments, n=21-29 junctions per co-transfection variant experiment.

SUPPLEMENTARY TABLE S1. Summary of *CELSR1* missense variants.

Sequence variant				Disease association		Reference
Cadherin Domain	Nucleotide	AA	gnomAD allele frequency	CHD	NTD	
3	c.1621C>T	p.R541W	8.68e-6		MMC	Allache et al., 2012
3	c.1651G>A	p.V551M	6.19e-7		LMC	Allache et al., 2012
4	c.1795C>T	p.R599W	1.18e-5	VSD, ASD, PDA, PFO		Qiao et al., 2016
5	c. 2141G>A	p.R714H‡	2.11e-4		AN, SB	Chen et al., 2018
5	c.2305C>T	p.R769W‡	6.33e-5		SB	Wang et al., 2018
5	c.2318C>T	p.A773V	9.21e-4		CRN	Robinson et al., 2012
6	c.2506C>T	p.R836C	6.20e-6		Lipoma	Allache et al., 2012
6	c.2609C>T	p.P870L	4.21e-5	PMVSD, PFO	CRN	Qiao et al., 2016
6	c. 2624C>T	p.T875I	9.73e-5		n.sp.	Chen et al., 2018
6	c.2644A>G	p.I882V	never seen	ASD		Qiao et al., 2016
8	c.3022G>C	p.V1008L	1.24e-6		TFT	Allache et al., 2012
8	c.3055G>T	p.A1019S	6.04e-3		n.sp.	Chen et al., 2018
8	c.3068C>G	p.A1023G	never seen		MMC	Lei et al., 2014
8	c.3257C>T	p.T1086M‡	1.01e-4		SB	Chen et al., 2018
9	c.3364G>A	p.G1122S‡	7.44e-6		AN	Wang et al., 2018
9	c.3372C>G	p.I1124M	7.44e-6		MMC	Lei et al., 2014
9	c.3562A>T	p.T1188S	9.31e-6		SB	Qiao et al., 2016

CHD (congenital heart defect): ASD, atrial septal defect; PDA, patent ductus arteriosus; PFO, paten foramen ovale; PMVSD, perimembranous ventricular septal defect; VSD, ventricular septal defect. **NTD (neural tube defect):** AN, anencephaly; CRN, craniorachischisis; LMC, lipomyelocele; MMC, myeleomeningocele; TFT, tight filum terminale; SB, spina bifida. AA, amino acid; n.sp., not specified; ‡denotes digenic variant

SUPPLEMENTARY TABLE S2. *In silico* predictions of CELSR1 missense variants identified in congenital heart and neural tube defect patients.

Cadherin Domain	Sequence variant		*In silico* predicted pathogenic effect							
	Human	Mouse	SIFT	PolyPhen-2	FATHMM (Inherited Disease)	FATHMM-XF	CADD	MutPred 2	Mutation Taster (2025)	Alpha Missense
3	R541W	R556W	++	++	++	++	++	++	++	-
3	V551M	V566M	++	++	++	++	++	++	++	++
4	R599W	R614W	-	++	-	++	+	++	++	-
5	R714H	R729H	-	++	++	-	+	-	++	-
5	R769W	R784W	++	++	-	++	+	++	++	-
5	A773V	A788V	++	+	-	-	-	-	++	-
6	R836C	R851C	++	++	++	-	+	-	++	-
6	P870L	P885L	-	++	-	++	++	++	++	++
6	T875I	T890I	-	+	-	++	+	++	++	-
6	I882V	I897V	-	-	-	-	-	-	++	-
8	V1008L	V1023L	-	++	-	++	+	++	++	+
8	A1019S	A1034S	-	++	++	++	+	-	-	-
8	A1023G	A1038G	++	+	-	++	+	++	++	++
8	T1086M	T1101M	-	++	++	++	+	++	++	-
9	G1122S	G1137S	++	++	-	++	+	-	++	-
9	I1124M	I1139M	++	++	++	-	-	++	++	-
9	T1188S	T1203S	-	-	-	-	-	-	-	-

Predictor outcomes:

SIFT	PolyPhen-2	FATHMM (Inherited Disease)	FATHMM-XF	CADD	MutPred2	MutationTaster (2025)	AlphaMissense
++ affected<br>- tolerated	++ probably damaging<br>+ possibly damaging<br>- benign	++ damaging<br>- tolerated	++ pathogenic<br>- benign	++ deleterious<br>+ possibly deleterious<br>- tolerated	++ pathogenic<br>+ benign	++ deleterious<br>- benign	++ likely pathogenic<br>+ ambiguous<br>- likely benign

SUPPLEMENTARY TABLE S3. Structural modeling of Celsr1 protein variants.

Cadherin Domain	Variant	Description of Structural Variation	AlphaFold2 Rendering of Variant Location
3	R556W	R = Basic (strong), polar, positively charged, hydrophilic<br>W = Basic (weak), polar, hydrophobic, aromatic, bulky<br>Arginine to Tryptophan between Cad2 and Cad3. The mutation from a polar, basic residue to an aromatic, bulky residue may alter dynamics of calcium binding.	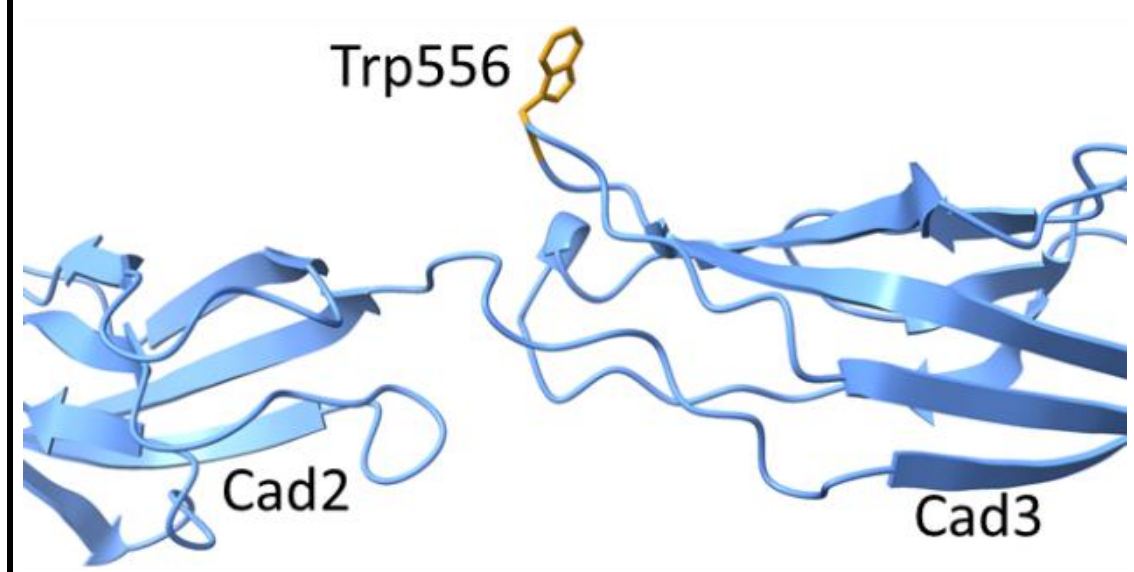
3	566M	V = Hydrophobic, nonpolar, aliphatic<br>M = Hydrophobic, sulfur-containing side chain, aliphatic<br>Valine to Methionine in β-sheet of Cad3. May alter local conformation but not expected to be significant.	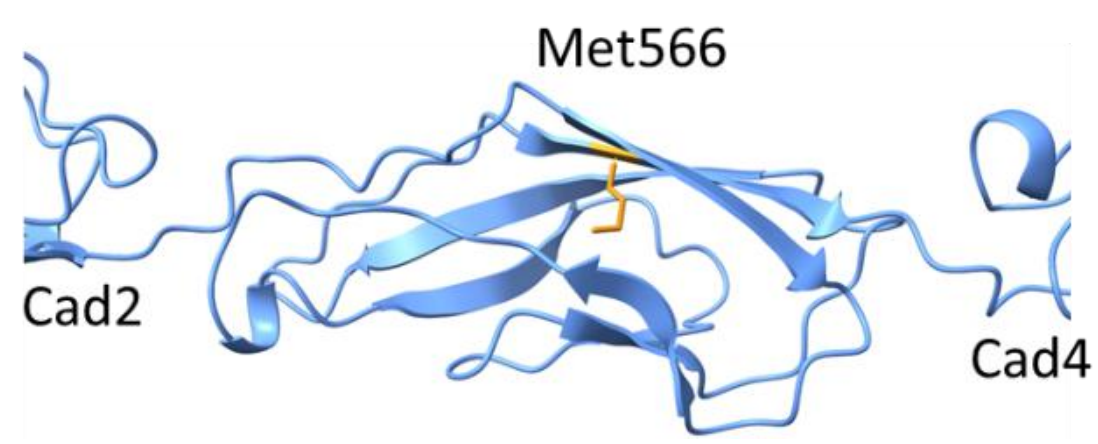
4	R614W	R = Basic (strong), polar, positively charged, hydrophilic<br>W = Basic (weak), polar, aromatic, bulky, hydrophobic<br>Arginine to Tryptophan neighboring β-sheet of Cad4. The mutation from a polar, basic residue to an aromatic, bulky may alter local conformation due to hydrophobicity.<br>Predicted phosphorylation site S631 located 17 residues downstream, thus may alter PTM through local changes in conformation but unlikely.	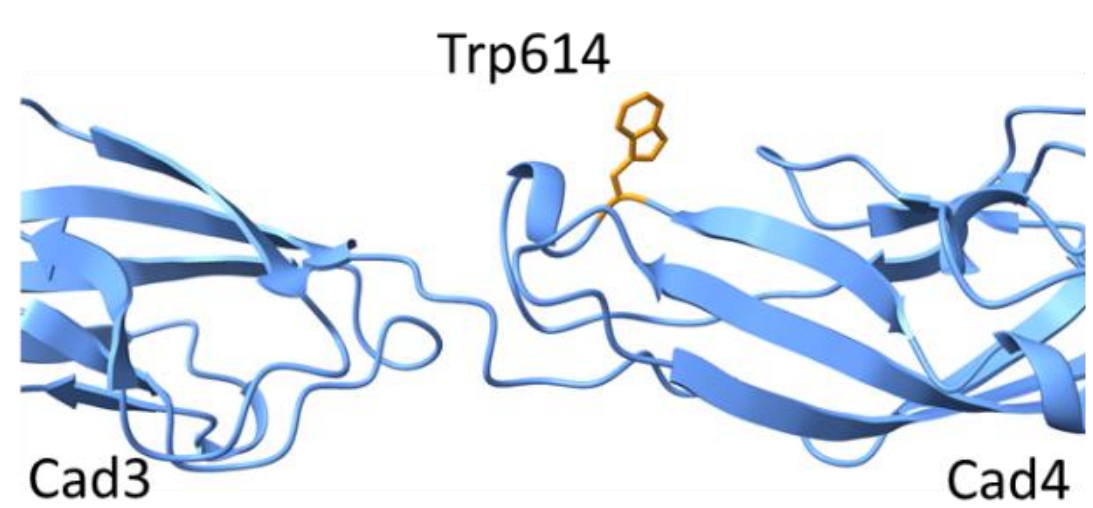
5	R729H	R = Basic (strong), polar, positively charged, hydrophilic<br>H = Basic, polar, aromatic, positively charged, hydrophobic<br>Arginine to Histidine near calcium binding regions. Due to similarities in side change properties, not expected to cause significant conformation changes.<br>Not expected to alter conformation nor PTMs, mutation is 34 residues from predicted phosphorylation site.	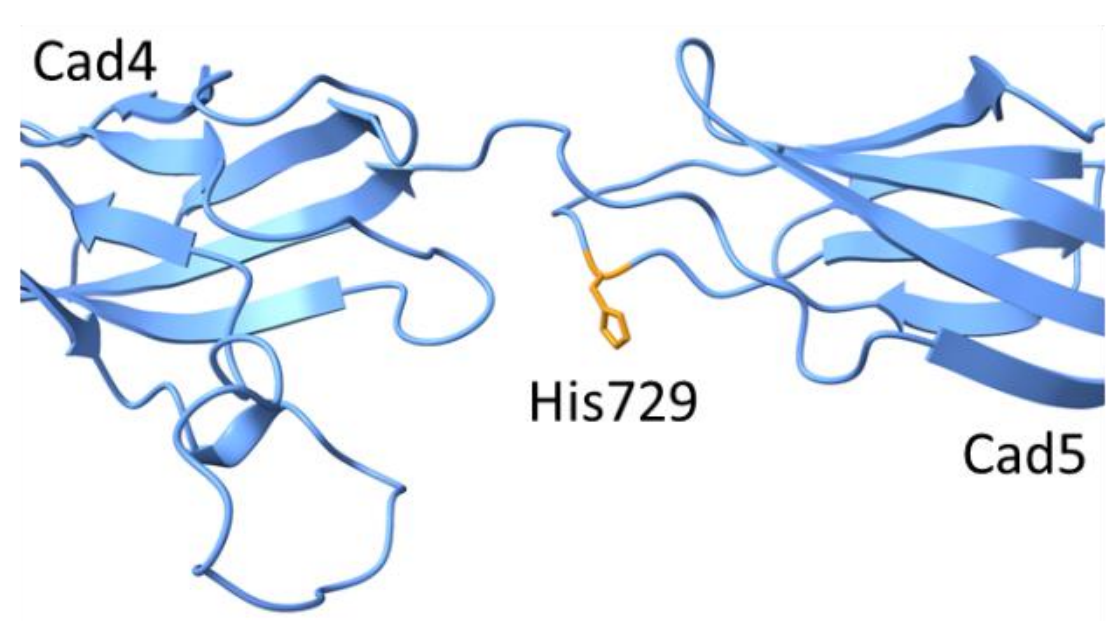

5	R784W	R = Basic (strong), polar, positively charged, hydrophilic<br>W = Basic (weak), polar, aromatic, bulky, hydrophobic<br>Arginine to Tryptophan in loop between β-sheet of Cad5, near calcium binding regions. Change from a polar, basic residue to an aromatic, bulky may alter local conformation due to hydrophobicity and effect calcium binding.<br>Not expected to alter conformation nor PTMs, mutation is 24 residues from predicted phosphorylation site.	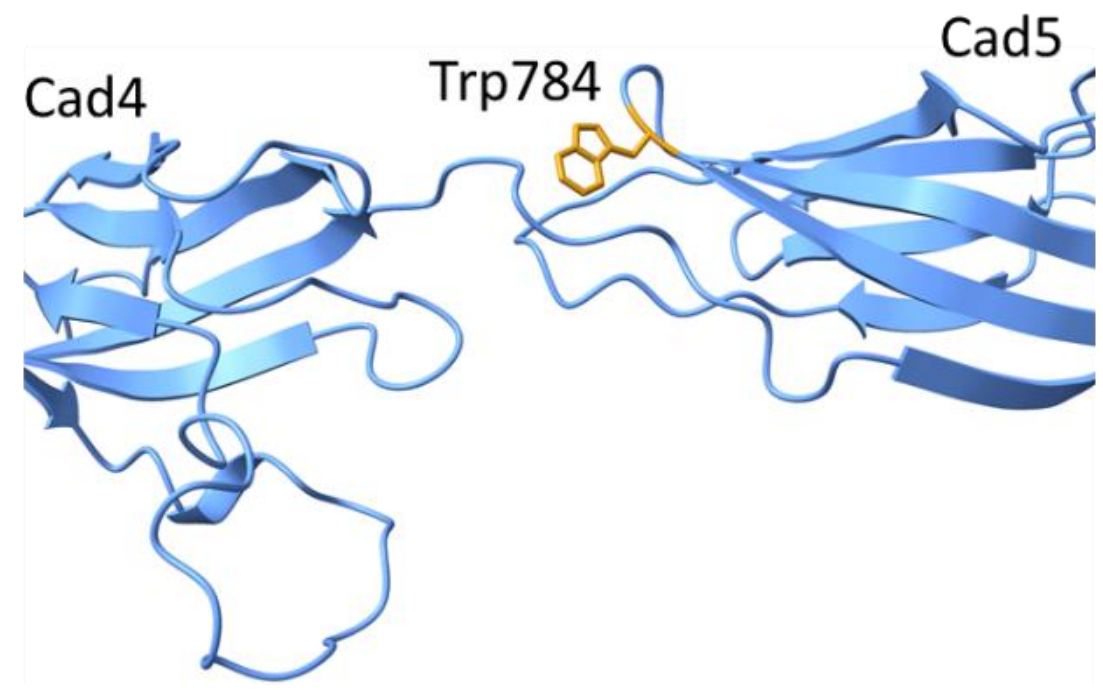
5	A788V	A = Hydrophobic, aliphatic, small<br>V = Hydrophobic, aliphatic<br>Alanine to Valine in β-sheet of Cad5. May alter local conformation but not expected to be significant due to side chain similarities.<br>Not expected to alter conformation nor PTMs, mutation is 28 residues from predicted phosphorylation site.	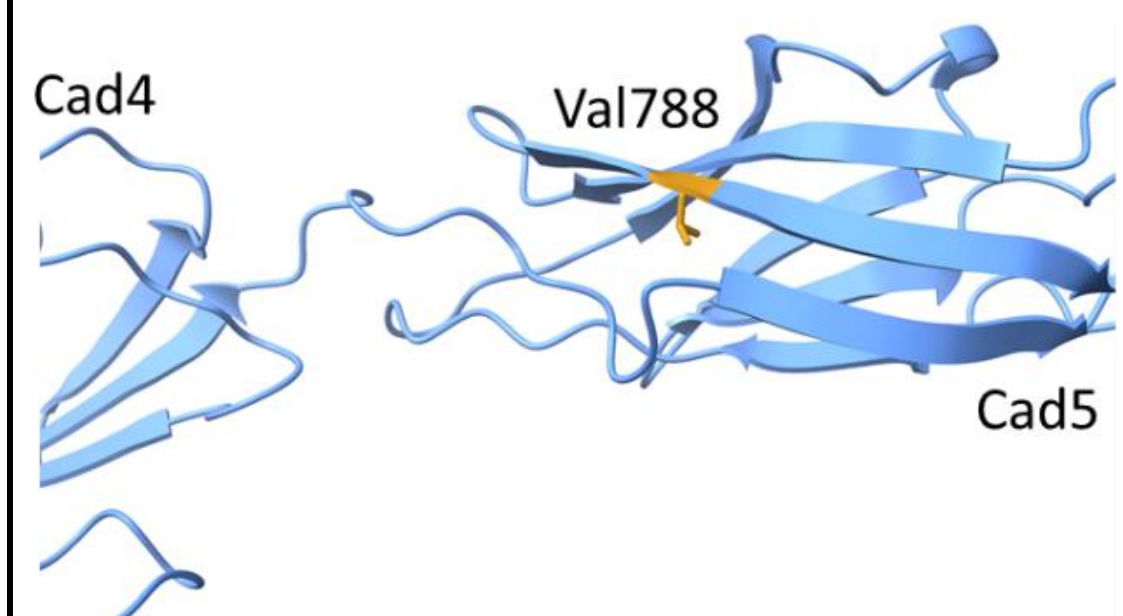
6	R851C	R = Basic (strong), polar, positively charged, hydrophilic<br>C = Nucleophilic, sulfur-containing side chain, hydrophobic, small<br>Arginine to Cysteine in β-sheet of Cad6. May disrupt local conformation due to change from large, basic side chain to small, nucleophilic side chain that may now form disulfide bonds.	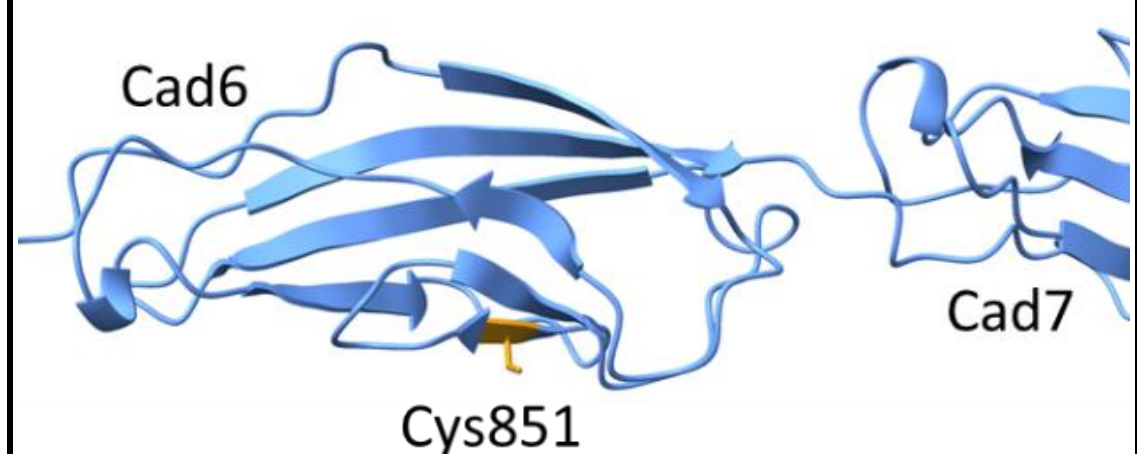
6	P885L	P = Hydrophobic, cyclic side chain, small<br>L = Hydrophobic, aliphatic<br>Proline to Leucine in loop between β-sheet of Cad6, near calcium binding regions. Change from small, cyclic side chain to larger aliphatic may alter local conformation and affect calcium binding.	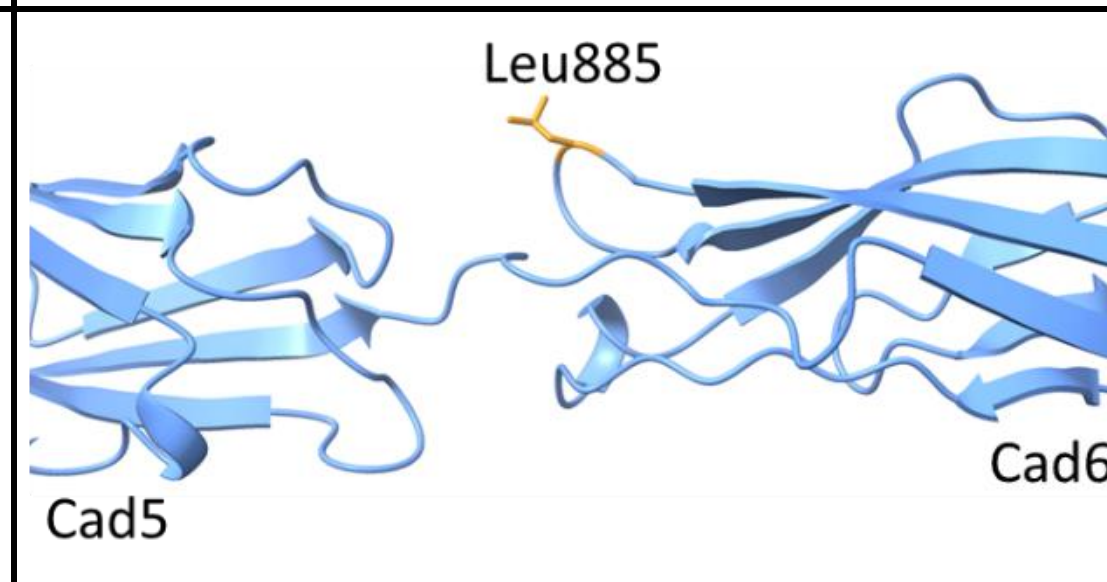

6	T890I	T = Nucleophilic, hydroxyl containing, hydrophobic, polar, small<br>I = Hydrophobic, aliphatic<br>Threonine to Isoleucine in β-sheet of Cad6. Change from small, nucleophilic side chain to larger aliphatic may alter local conformation or β-sheet.	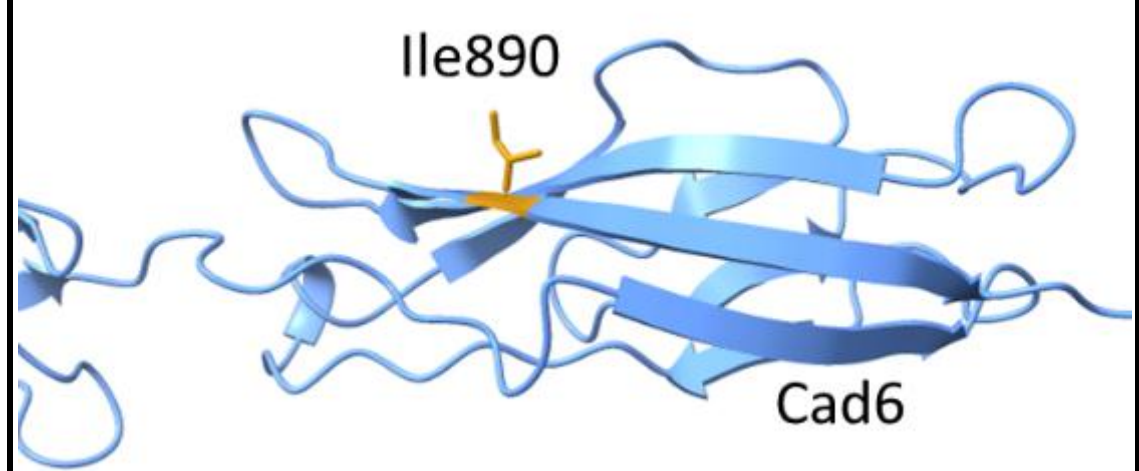
6	I897V	I = Hydrophobic, nonpolar, aliphatic<br>V = Hydrophobic, nonpolar, aliphatic<br>Isoleucine to Valine near calcium binding regions. Not expected to cause changes in conformation due to side chain similarities.	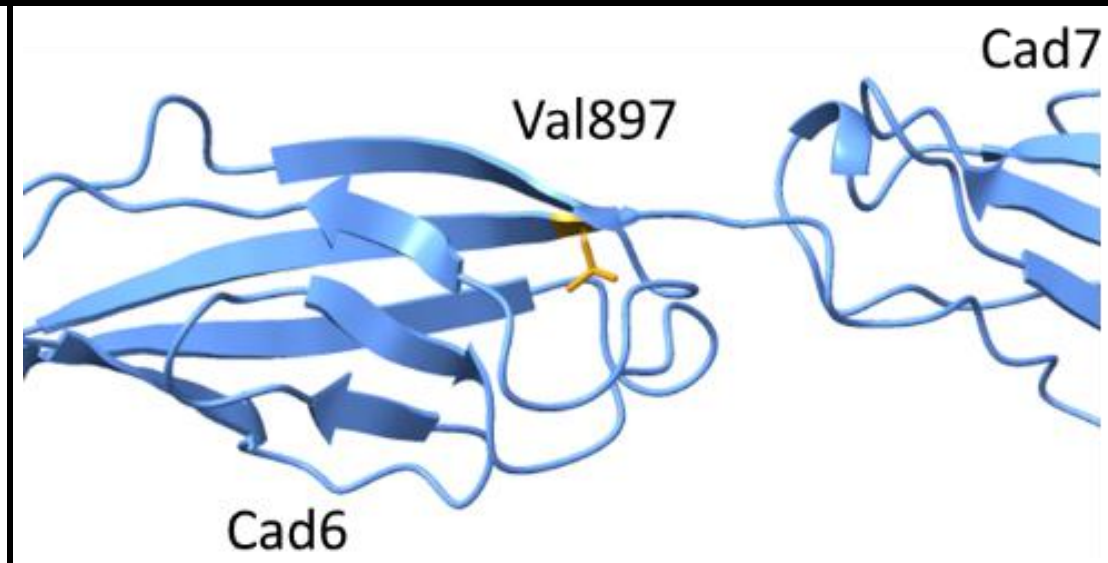
8	V1023L	V = Hydrophobic, nonpolar, aliphatic<br>L = Hydrophobic, nonpolar, aliphatic<br>Valine to Leucine in β-sheet of Cad8. Not expected to cause changes in conformation due to side chain similarities.	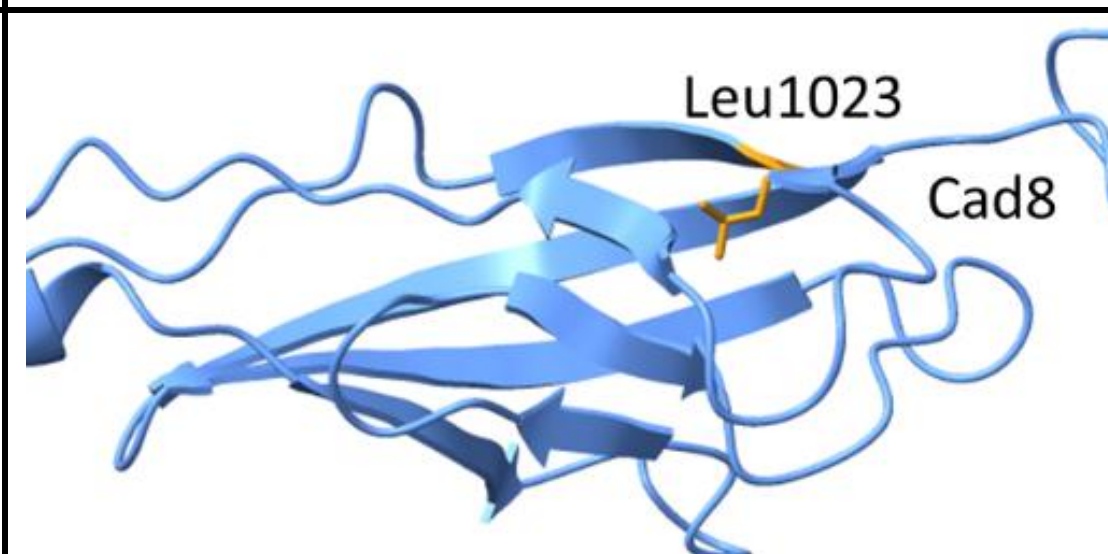
8	A1034S	A = Nonpolar, aliphatic, small, hydrophobic,<br>S = Nucleophilic, hydroxyl containing, polar, small, hydrophilic<br>Alanine to Serine in β-sheet of Cad8. May disrupt local conformation due to change from hydrophobic to nucleophilic side chain.	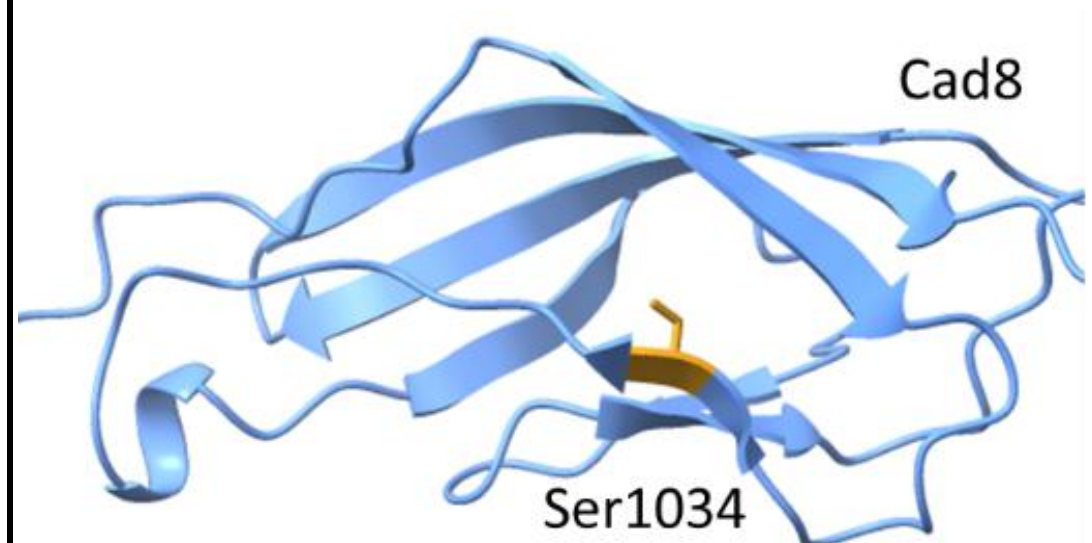
8	A1038G	A = Small, nonpolar, aliphatic, hydrophobic<br>G = Small, nonpolar, aliphatic, hydrophobic<br>Alanine to Glycine in Cad8. Not expected to cause changes in conformation due to side chain similarities.	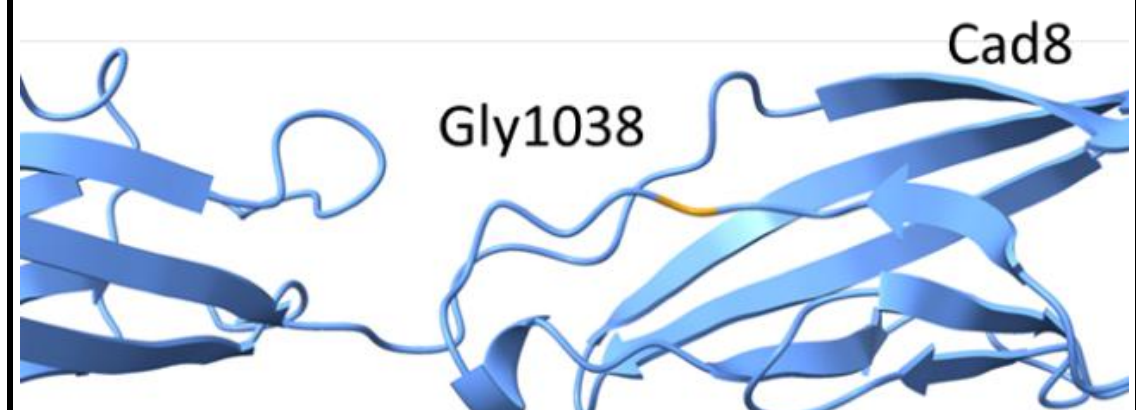

<table>
<tr>
<td>8</td>
<td>T1101M</td>
<td>T = Nucleophilic, polar, hydroxyl containing, hydrophobic, small

M = Nonpolar, sulfur-containing side chain, hydrophobic

Threonine to Methionine in β-sheet of Cad8. Not expected to cause significant change in conformation.</td>
<td>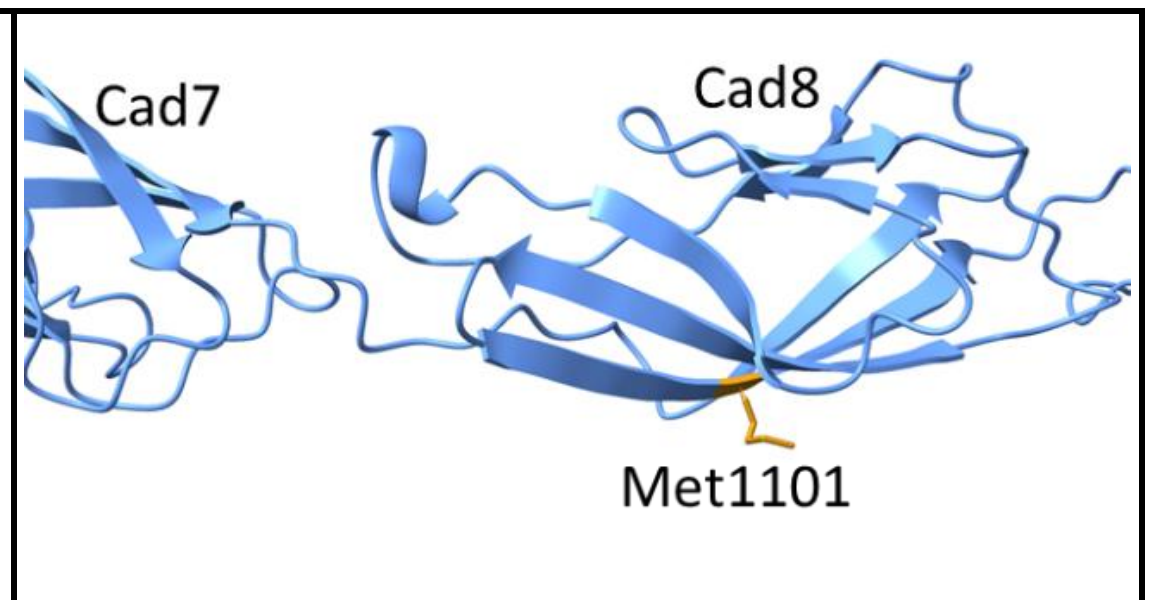
</td>
</tr>
<tr>
<td>9</td>
<td>G1137S</td>
<td>G = Small, nonpolar, aliphatic, hydrophobic

S = Nucleophilic, polar, hydroxyl containing, small

Glycine to Serine in Cad9. Expected to cause changes in local conformation due change from hydrophobic to nucleophilic side chain.</td>
<td>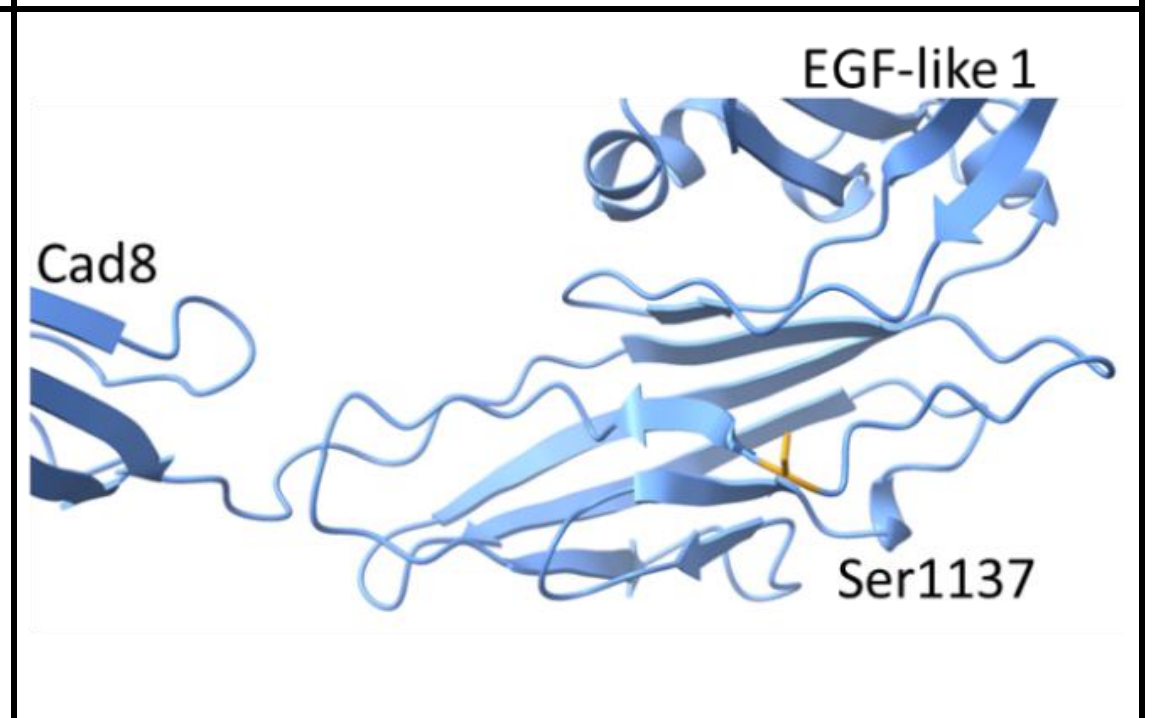
</td>
</tr>
<tr>
<td>9</td>
<td>I1139M</td>
<td>I =Hydrophobic, nonpolar aliphatic

M = Hydrophobic, nonpolar, sulfur-containing side chain

Isoleucine to Methionine in β-sheet of Cad9. Not expected to cause significant change in conformation due to side chain similarities.</td>
<td>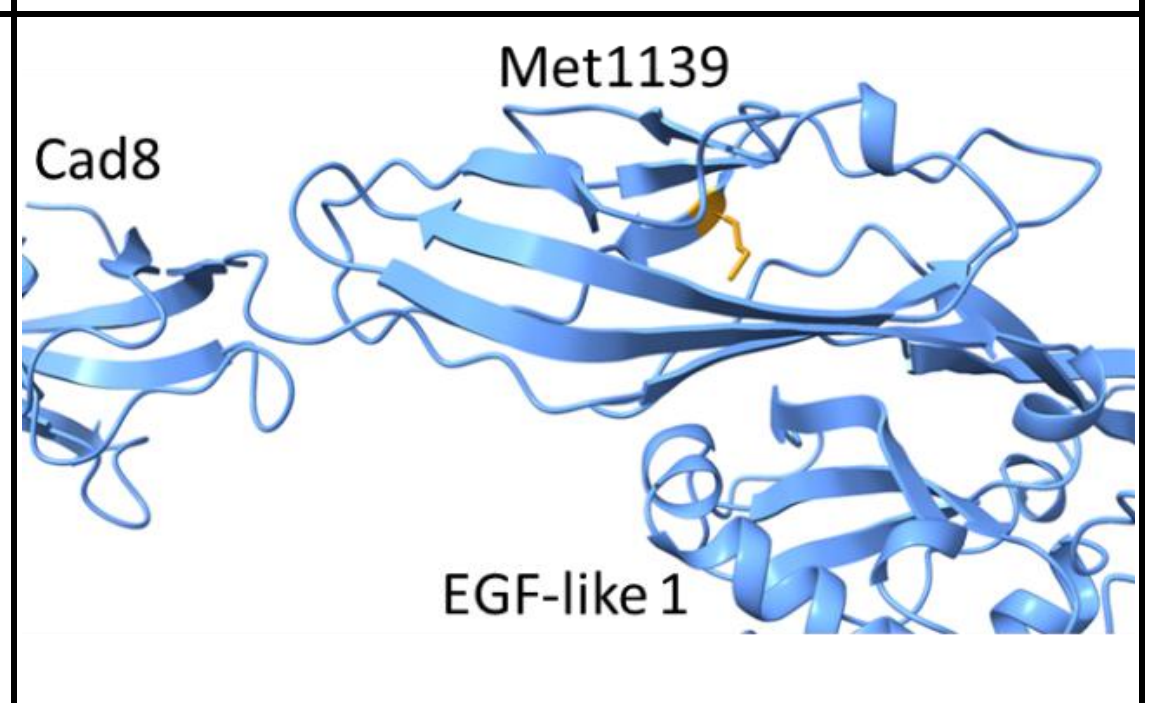
</td>
</tr>
<tr>
<td>9</td>
<td>T1203S</td>
<td>T = Nucleophilic, hydroxyl containing, hydrophobic, polar, small

S = Nucleophilic, hydroxyl containing, polar, small

Threonine to Serine in β-sheet of Cad9. Not expected to cause conformation changes due to side chain similarities.</td>
<td>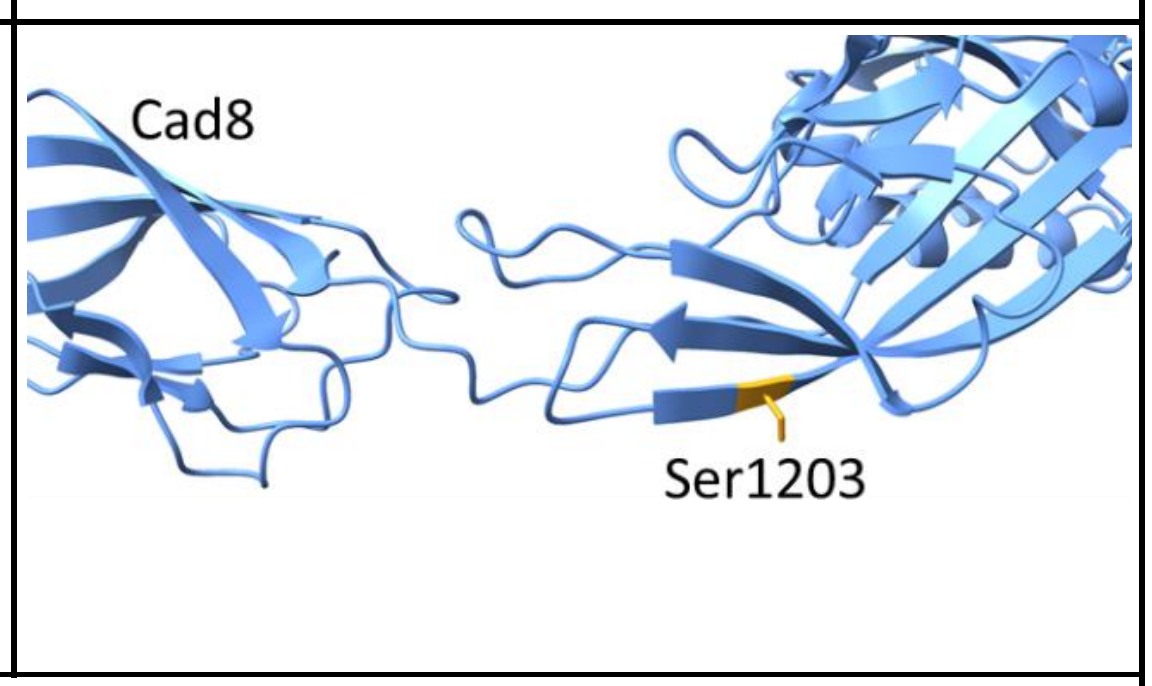
</td>
</tr>
<tr>
<td colspan="4">Description of resulting amino acid change and any predicted property or conformational changes introduced by indicated mutations. Disease-associated residue (colored yellow) locations within AlphaFold2-predicted protein structure of mouse Celsr1. Mouse-equivalent residues of disease-associated human mutations are shown. Unless otherwise indicated, changes to post-translational modifications (PTMs) are not expected.</td>
</tr>
</table>

SUPPLEMENTARY TABLE S4. Celsr1 variant proximity to nearest calcium ion.

Human variant	Mouse equivalent	EC domain	Present in 8D40[1]	Present in 7SZ8[2]	Present in 8VY2[3]	Proximity to calcium ion in 8D40 (Å)	Proximity to calcium ion in 7SZ8 (Å)
R541W	R556W	EC3	Yes	No	No	10.0	
V551M	V566M	EC3	Yes	No	No	22.1	
R599W	R614W	EC4	Yes	Yes	No	8.2	7.6
R714H	R729H	EC5	No	Yes	No		6.6
R769W	R784W	EC5	No	Yes	No		6.3
A773V	A788V	EC5	No	Yes	No		16.3
R836C	R851C	EC6	No	Yes	No		21.0
P870L	P885L	EC6	No	Yes	No		9.2
T875I	T890I	EC6	No	Yes	No		13.9
I882V	I897V	EC6	No	Yes	No		9.8
V1008L	V1023L	EC8	No	No	No		
A1019S	A1034S	EC8	No	No	No		
A1023G	A1038G	EC8	No	No	No		
T1086M	T1101M	EC8	No	No	No		
G1122S	G1137S	EC9	No	No	Yes		
I1124M	I1139M	EC9	No	No	Yes		
T1188S	T1203S	EC9	No	No	Yes		

[1]PDB ID code – 8D40 (http://doi.org/10.2210/pdb8D40/pdb)
[2]PDB ID code – 7SZ8 (http://doi.org/10.2210/pdb7SZ8/pdb)
[3]PDB IC code – 8VY2 (http://doi.org/10.2210/pdb8VY2/pdb)
